# *Naja atra* SVPLA_2_ contributes to venom-induced AKI via HRAS palmitoylation-dependent PI3K-AKT inactivation

**DOI:** 10.1016/j.jlr.2026.101123

**Published:** 2026-08-14

**Authors:** Jiahao Liu, Sitong Chen, Jiajia Wu, Sunkun Tang, Zejing Wen, Xiaowen Bi, Yang Yang, Chunhong Huang

**Affiliations:** 1 School of Basic Medical Sciences, Jiangxi Medical College, Nanchang University, Nanchang, China; 2 The First School of Clinical Medicine, Jiangxi Medical College, Nanchang University, Nanchang, China; 3 The Second School of Clinical Medicine, Jiangxi Medical College, Nanchang University, Nanchang, China; 4 The Second Affiliated Hospital, Jiangxi Medical College, Nanchang University, Nanchang, China

**Keywords:** lipid rafts, palmitoylation, phospholipase/A_2_, phospholipids/metabolism, apoptosis, snake venom, *Naja atra*, acute kidney injury, PI3K–AKT, hexokinase 2

## Abstract

Clinically, acute kidney injury (AKI) is one of the most frequent complications of *Naja atra* (*N. atra*) envenomation, primarily attributed to snake venom phospholipase A_2_ (SVPLA_2_). Although the SVPLA_2_ inhibitor varespladib shows great therapeutic promise, the underlying mechanisms remain incompletely understood. Herein, we integrated multi-omics and molecular biology approaches to investigate the critical role of SVPLA_2_ in *N. atra* venom-induced AKI as evidenced by pharmacological inhibition with varespladib. Proteomic profiling identified HRAS and CCND1 as key mediators of SVPLA_2_-induced nephrotoxicity. Mechanistically, SVPLA_2_ disrupts lipid raft integrity, impairing HRAS palmitoylation–dependent plasma membrane localization and GTPase activity. This defect suppresses phosphatidylinositol 3-kinase (PI3K)/protein kinase B (AKT) signaling, causing apoptosis and cell cycle arrest in renal tubular epithelial cells, enforced activation of PI3K–AKT signaling effectively rescued cells from injury. Beyond direct cytotoxicity, SVPLA_2_ profoundly remodels the renal immune microenvironment. SVPLA_2_ enhances glycolysis by upregulating hexokinase 2 (HK2) while suppressing fatty acid oxidation through downregulation of carnitine palmitoyltransferase IA, thereby metabolically driving M1 polarization. This metabolic shift impairs macrophage efferocytosis and sustains inflammatory injury. HK2 knockdown reverses these effects in macrophages. Taken together, this study reveals a dual epithelial–immune mechanism by which SVPLA_2_ activity critically contributes to *N. atra* venom-induced AKI, as evidenced by pharmacological inhibition with varespladib. These findings highlight PI3K–AKT signaling and HK2 as potential therapeutic targets for *N. atra*-triggered AKI.

Snakebite represents a significant yet neglected global challenge, characterized by high morbidity, mortality, and wide geographical distribution (1, 2, 3). A major obstacle to reducing its burden is the incomplete understanding of venom-induced pathophysiology, which hinders the development of novel targeted drugs for severe complications.

In southern China, envenomation by *Naja atra* (*N. atra*) represents a medical emergency (4). Despite timely antivenom administration, the venom can still cause local necrosis and persistent organ dysfunction. Among these, acute kidney injury (AKI) is the most severe and frequent complication (5, 6, 7, 8, 9, 10). Accumulating evidence indicates that snake venom phospholipase A_2_ (SVPLA_2_) is the major toxin responsible for inducing muscle necrosis and organ damage (7, 8, 9, 10). The venom composition of *N. atra* is complex and varies considerably depending on multiple factors, including geographical origin, sex, age, and captive versus wild habitat (11, 12). Nevertheless, two major toxin families consistently dominate the venom proteome: three-finger toxins (3FTxs) and SVPLA_2_. 3FTxs are widely recognized as the most abundant components, with reported proportions ranging from approximately 40% to over 84% of total venom proteins, whereas SVPLA_2_ constitutes a variable, with reported values ranging from approximately 12%–46% depending on the venom source (11, 12, 13, 14, 15, 16). Although SVPLA_2_ is not necessarily the most abundant toxin in *N. atra* venom, its pharmacological inhibition substantially attenuates venom-induced pathology (7, 8, 10). This raises the possibility that SVPLA_2_ may act in a synergistic manner with other venom components, such as 3FTxs, rather than functioning as a quantitatively dominant toxin. Therefore, understanding the pathogenic role of SVPLA_2_ is of particular importance for elucidating the mechanisms of *N. atra* envenomation and developing targeted therapeutic strategies.

Phospholipase A_2_ (PLA_2_) enzymes are classified into acidic and basic isoforms. Acidic isoforms induce postsynaptic neurotoxicity via non-enzymatic receptor binding, whereas basic isoforms exert presynaptic neurotoxicity, cytotoxicity and myotoxicity through their hydrolytic activity. *N. atra* SVPLA_2_ is predominantly a basic isoform, which not only damages local tissues but also modulates immune cell functions (10, 11, 17, 18). Given the limitations of antivenom, including high species-specificity (limited to a few snake species), allergic reactions, and poor accessibility, small-molecule inhibitors have attracted increasing interest. Varespladib, a specific PLA_2_ inhibitor, neutralizes SVPLA_2_ activity and serves as both a potential broad-spectrum antidote and a valuable pharmacological tool (19, 20, 21, 22, 23).

To elucidate the molecular basis of *N. atra* SVPLA_2_-induced AKI, quantitative proteomic analysis of murine renal tissue was conducted, which identified HRAS and CCND1 as key mediators. Gene Ontology (GO) analysis showed significant enrichment in GTPase activity, GTPase activator activity, and G1/S cell cycle transition. In contrast, Kyoto Encyclopedia of Genes and Genomes (KEGG) enrichment highlighted dysregulation of phosphatidylinositol 3-kinase (PI3K)/protein kinase B (AKT), metabolic, and Ras signaling pathways, collectively indicating disruption of cellular survival, proliferation, and metabolic homeostasis. HRAS acts as a canonical molecular switch governing PI3K–AKT signaling and cell fate decisions, processes critically implicated in renal tubular injury and maladaptive repair (24, 25). CCND1 centrally regulates G1/S transition, and its aberrant activation is linked to pathological cell cycle re-entry, tubular epithelial dysfunction, and progressive kidney injury (26). Particularly, HRAS is a small GTPase of the RAS family whose proper localization is essential for downstream signal transduction. Numerous studies have demonstrated that palmitoylation is a common and major post-translational modification of HRAS, enabling its stable anchoring to lipid microdomains (27, 28, 29). This modification is reversibly regulated by palmitoyl acyltransferases and depalmitoylating enzymes. In addition to enzymatic regulation, the integrity of lipid rafts is critical for the functional compartmentalization of HRAS (30, 31). Disruption of HRAS palmitoylation or its membrane microenvironment leads to aberrant downstream signaling (32, 33). Notably, although SVPLA_2_ hydrolyzes the sn-2 ester bond of glycerophospholipids within the plasma membrane (34), whether it disrupts lipid raft integrity to interfere with HRAS palmitoylation, and whether the resulting aberrant HRAS signaling affects downstream PI3K–AKT pathway activation, remain unknown.

Beyond direct cytotoxicity, AKI pathogenesis is profoundly shaped by the renal immune microenvironment (35, 36, 37), and the effect of snake venom toxin on macrophages has garnered widespread attention (38, 39, 40). The functional plasticity of macrophages is closely linked to alterations in their metabolic programming. Aerobic glycolysis is the primary metabolic pathway driving proinflammatory responses in classically activated (M1) macrophages (41, 42). Our previous studies have demonstrated that *N. atra* SVPLA_2_ drives a metabolic shift in bone marrow-derived macrophages (BMDMs), characterized by enhanced glycolytic signatures, a bias toward the M1 proinflammatory phenotype, and impaired efferocytic capacity, which may be associated with the abnormal upregulation of pyruvate kinase M2 (PKM2) and hexokinase 2 (HK2), which are rate-limiting enzymes catalyzing irreversible reactions in glycolysis (43). However, that study did not perform an in-depth analysis of cellular energy metabolism, nor did it evaluate the metabolic pathways fatty acid oxidation (FAO) and oxidative phosphorylation associated with M2 macrophages, which are primarily responsible for tissue repair and efferocytosis (41, 42). Therefore, this study aims to fill these gaps and to determine whether targeting specific metabolic enzymes can rescue the induced metabolic imbalance in macrophages triggered by SVPLA_2_.

Taken together, our findings confirm that targeted SVPLA_2_ inhibition with varespladib confers marked protection against *N. atra* venom-induced AKI while also revealing the pivotal role of SVPLA_2_ in mediating the venom’s renal toxicity via a pharmacological dissection strategy. Our findings extend the conventional paradigm of snake venom toxicity by revealing an integrated link between membrane structural disruption, post-translational modification, signaling inactivation, and immune dysfunction, thereby providing a mechanistic rationale for targeting SVPLA_2_-dependent membrane signaling and immune reprogramming in the treatment of venom-induced AKI.

## Materials and Methods

### Animal experiment and ethics

Animal procedures complied with the ARRIVE guidelines and were approved by the Animal Care and Use Committee of Nanchang University (NCULAE-20220624042). Six-week-old C57BL/6J mice (20 ± 3 g) were randomly divided into four groups (n = 5): (a) NS group: mice received intraperitoneal (i.p.) injection of normal saline (100 μl), followed 20 min later by tail vein (i.v.) injection of normal saline (100 μl); (b) DMSO + Var group: mice received i.p. injection of normal saline (100 μl), followed 20 min later by i.v. injection of varespladib (1.75 mg/kg in 100 μl DMSO/saline solution); (c) NA group: mice received i.p. injection of *N. atra* venom (0.47 mg/kg, 0.5 LD_50_, in 100 μl normal saline), followed 20 min later by i.v. injection of normal saline (100 μl); (d) NA + Var group: mice received i.p. injection of *N. atra* venom (0.47 mg/kg, 0.5 LD_50_, in 100 μl normal saline), followed 20 min later by i.v. injection of varespladib (1.75 mg/kg in 100 μl DMSO/saline solution).

Varespladib was dissolved in DMSO to prepare a 5 mg/ml stock solution. This stock was then diluted with sterile normal saline to a final working concentration of 350 μg/ml. The 20-min interval between venom and varespladib administration was designed to mimic a real-world clinical treatment scenario. All mice were euthanized at 12 h post-venom injection, and all animals survived to this endpoint. EDTA-anticoagulated blood samples were collected via cardiac puncture, and kidneys were harvested for subsequent analyses.

### Cell culture and mycoplasma monitoring

RAW 264.7 (murine macrophage-like cells) (RRID: CVCL_0493; Cat. No. IM-M028) and HK-2 cells (human renal proximal tubular epithelial cells) (RRID: CVCL_0302; Cat. No. IM-H060) were obtained from Immocell Biotechnology (Xiamen, China). BMDMs were isolated from mice and differentiated under standard macrophage-inducing conditions. Moreover, to ensure data rigor and reproducibility, all cell lines were maintained at a low passage number (below 10). Mycoplasma contamination was routinely monitored at each passage and during every freeze-thaw cycle, with all tests returning negative results.

### Statistical analysis

All data are expressed as mean ± SD. For in vivo experiments, each group consisted of five mice (n = 5, independent biological replicates). For in vitro studies, each condition was tested using three independent biological replicates (n = 3). The Shapiro–Wilk test was used to assess normality, and the Brown-Forsythe test to evaluate homogeneity of variances. Comparisons involving multiple groups with normally distributed and homoscedastic data were performed using ANOVA followed by Tukey’s post hoc test. Non-normally distributed data were compared using the Kruskal–Wallis test with Dunn’s multiple comparison procedure. A *P* value < 0.05 was considered statistically significant. GraphPad Prism 9.0 was employed for all statistical analyses.

### Other methodological information

Other detailed methodological information is provided in Supplementary material 1. The representative western blotting images are available in Supplementary Material 2.

## Results

### *N. atra* SVPLA_2_ induces AKI in mice

**Fig. 1:**
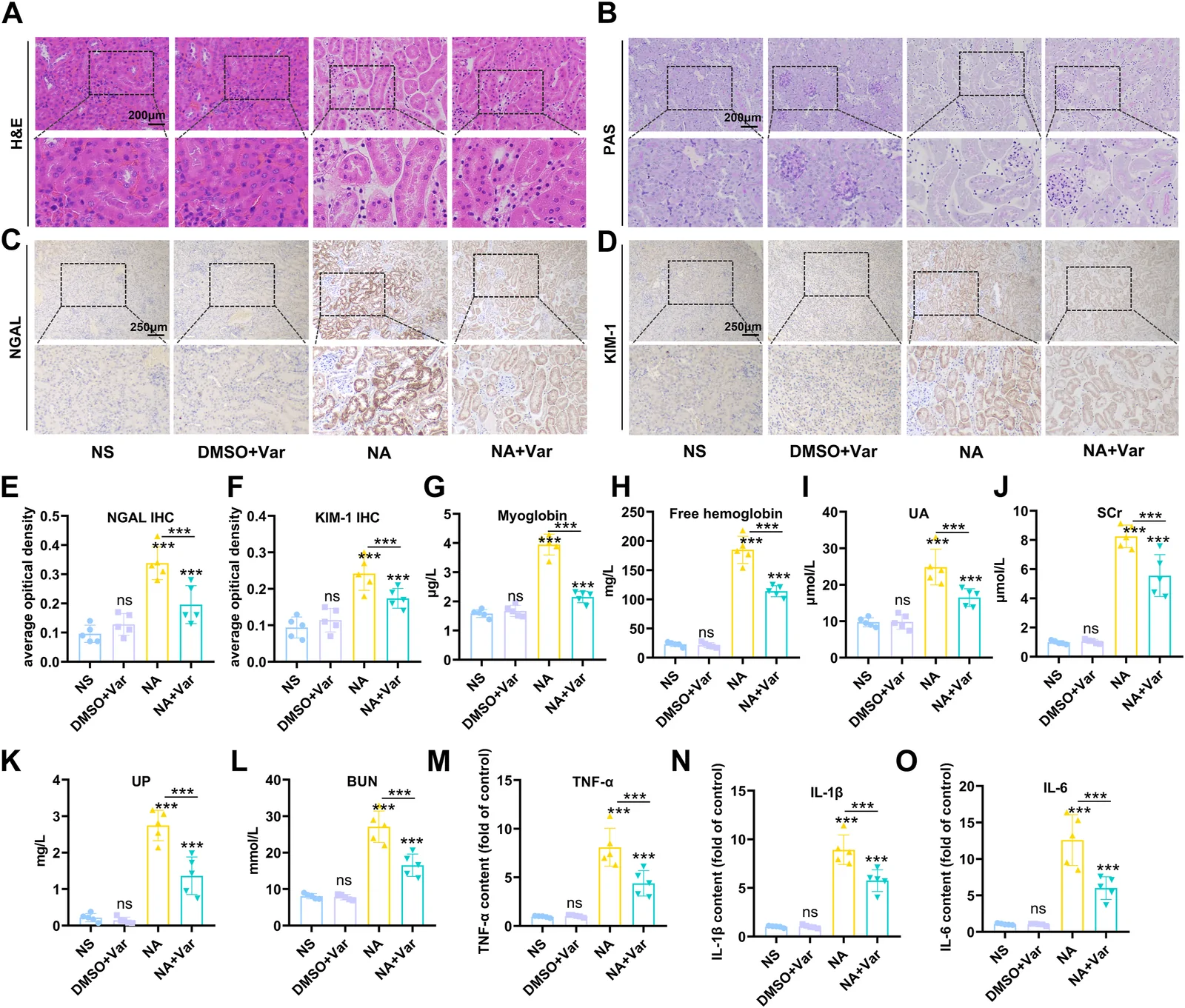
*N. atra* SVPLA_2_ drives AKI in mice. A, B: Renal histopathology assessed by H&E and PAS staining. C, D: IHC analysis of NGAL and KIM-1 in kidney sections. E, F: Quantification of IHC staining intensity. G, H: Detection of Myoglobin and free hemoglobin. I–L: Assessment of renal function parameters, including UA, SCr, UP, and BUN. (M–O) Renal levels of pro-inflammatory cytokines TNF-α, IL-1β, and IL-6. All data are shown as mean ± SD. Significance levels: ∗∗∗*P* < 0.001. ns denotes not significant versus the specified group indicated group. n = 5 (independent biological replicates). The scale bar is marked in the image.

On hematoxylin and eosin (H&E) staining, the injured kidneys exhibited swollen tubular epithelial cells, dilated lumina, proteinaceous casts, expanded interstitium, and patchy necrosis. Periodic acid Schiff (PAS) staining further showed pronounced loss of the brush border and depletion of glycogen stores in the tubular epithelium. These pathological changes were markedly attenuated by treatment with varespladib (Fig. 1A, B). Correspondingly, immunohistochemistry (IHC) revealed strong upregulation of both renal tubular injury markers—NGAL and KIM-1—in the NA group, which was significantly reduced in the NA + Var group (Fig. 1C–F) (44). *N. atra* venom exposure significantly elevated serum myoglobin and free hemoglobin levels, effects that were reversed by varespladib treatment (Fig. 1G, H). Moreover, *N. atra* venom caused pronounced renal dysfunction, as indicated by elevated levels of serum uric acid (UA), creatinine (SCr), blood urea nitrogen (BUN), and urine protein (UP) (Fig. 1I–L). Additionally, venom-induced AKI was accompanied by increased renal levels of common cellular inflammatory factors, all of which were suppressed upon varespladib (Fig. 1M–O). These findings indicate that SVPLA_2_ is a key driver of *N. atra* venom–induced AKI.

### Proteomics identifies HRAS and CCND1 as central targets of SVPLA_2_-induced nephrotoxicity

**Fig. 2:**
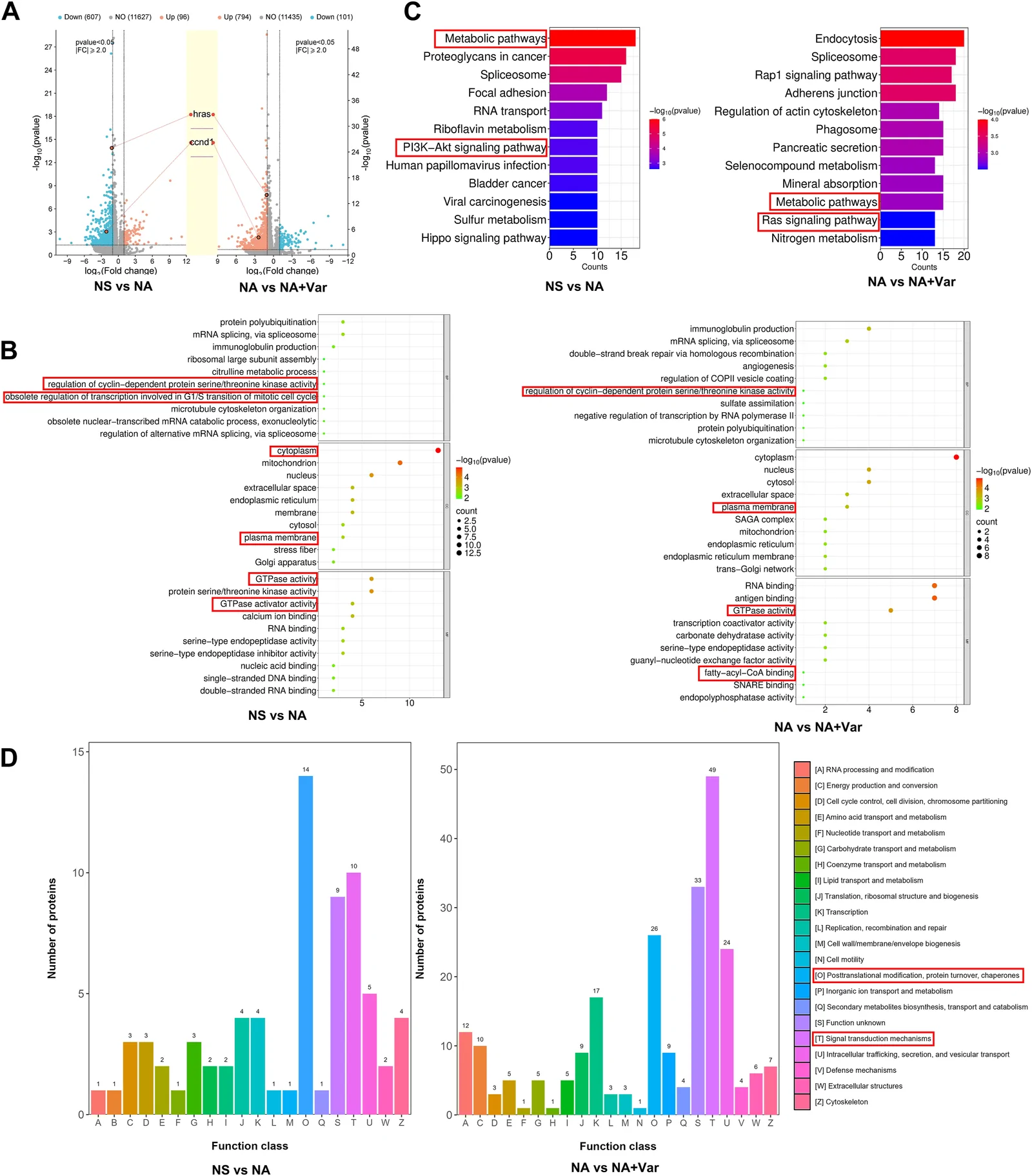
Proteomic and bioinformatic analysis in SVPLA_2_-induced nephrotoxicity. A: Volcano plots of DEPs. B: GO analysis. C: KEGG analysis. D: COG analysis. n = 6 (independent biological replicates).

To explore potential toxic targets and mechanisms of *N. atra* SVPLA_2_-caused nephrotoxicity, quantitative proteomic profiling of mouse renal tissues (NS, NA, and NA + Var groups) was performed. Differential proteins were subjected to dual-algorithm analysis (Mcode and cytoHubba), and the intersection with the highest scores identified HRAS and CCND1 as core targets (Supplementary Table S5 in Supplementary material 1). GO and KEGG enrichment analyses revealed that SVPLA_2_-induced proteomic changes were significantly enriched in GTPase activity, cell cycle regulation, PI3K–AKT signaling, and metabolic pathways, all of which were markedly attenuated by varespladib (Fig. 2B, C). Notably, these results are highly interrelated, as HRAS functions as an upstream small GTPase that directly couples membrane-associated signaling to PI3K–AKT pathway activation, positioning it as a plausible molecular link between SVPLA_2_-induced proteomic changes and downstream signaling. Moreover, functional annotation using Clusters of Orthologous Groups (COG) classification showed that category O (Post-translational modification, protein turnover, chaperones) was significantly upregulated (Fig. 2D), highlighting post-translational regulation may be an important layer affected by SVPLA_2_ exposure.

### SVPLA_2_ impairs HRAS membrane localization and suppresses GTPase activity

**Fig. 3:**
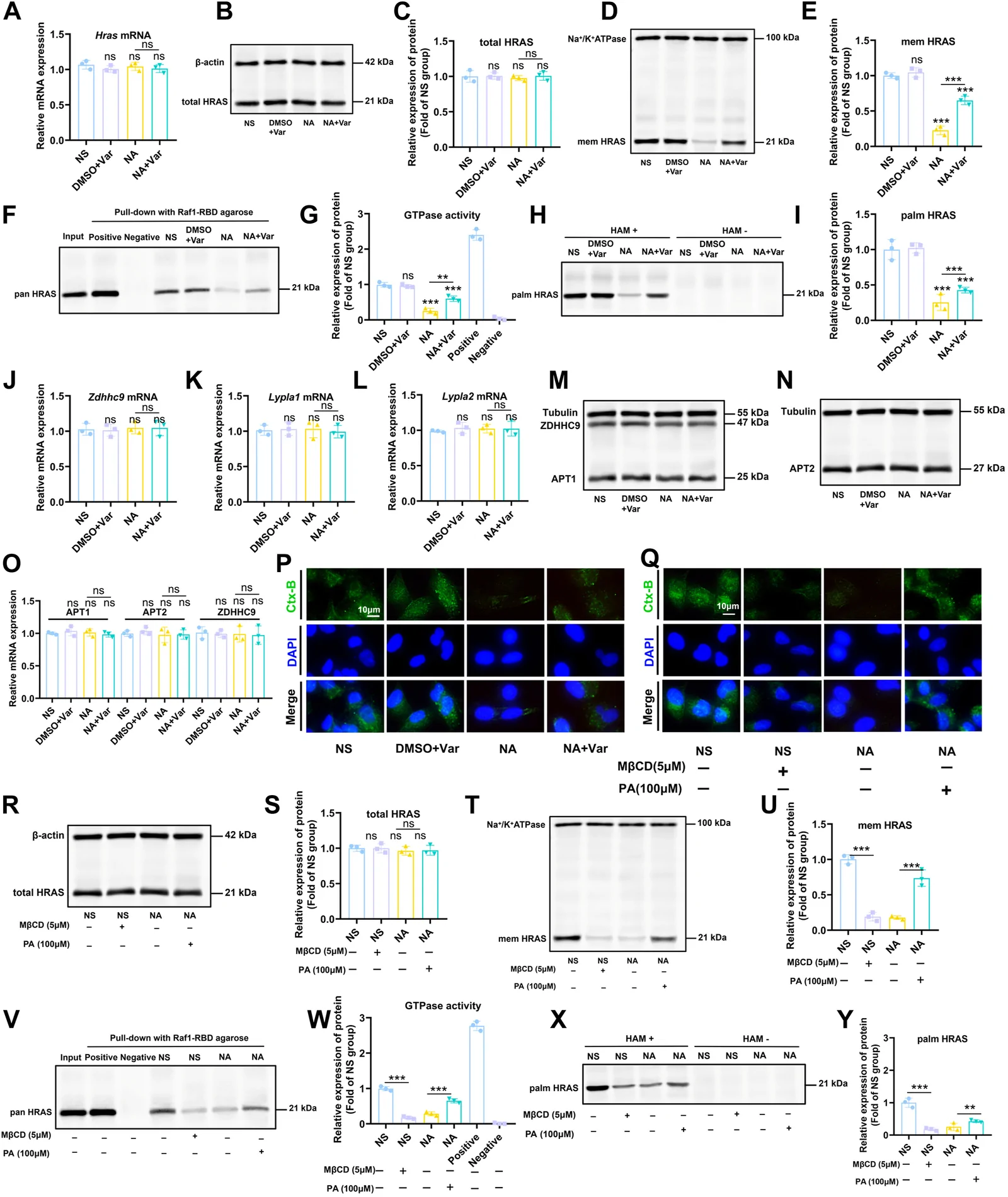
SVPLA_2_ disrupts HRAS membrane localization and GTPase activity by impairing lipid raft-dependent palmitoylation. A–C: RT–PCR and immunoblot analyses of *Hras* mRNA and total HRAS protein levels following venom exposure. D, E: Immunoblot analysis and quantification of membrane-associated HRAS after subcellular fractionation. F, G: Active HRAS pull-down assays demonstrating reduced GTP-bound HRAS activity in venom-treated samples. H, I: Acyl–biotin exchange assays assessing HRAS palmitoylation levels. J–O: RT–PCR and immunoblot analyses of palmitoylation-modifying enzymes ZDHHC9, APT1, and APT2. P: Confocal microscopy of lipid rafts visualized by Ctx-B staining. R–Y: Effects of lipid raft depletion and PA supplementation on lipid raft integrity, HRAS membrane localization, HRAS palmitoylation, and HRAS GTPase activity. All data are shown as mean ± SD. Significance levels: ∗∗*P* < 0.01, ∗∗∗*P* < 0.001. ns denotes not significant versus the specified group indicated group. n = 3 (independent biological replicates). The scale bar is marked in the image.

We first examined whether SVPLA_2_ alters HRAS expression. Interestingly, neither *Hras* mRNA levels nor total protein abundance showed significant differences among groups (Fig. 3A–C), indicating that SVPLA_2_ does not regulate HRAS at the transcriptional or total protein level. Since HRAS signaling activity critically depends on its plasma membrane localization, we further assessed membrane-associated HRAS. Subcellular fractionation revealed a marked reduction of membrane-bound HRAS in SVPLA_2_-treated HK-2 cells (Fig. 3D, E). Thus, SVPLA_2_ selectively disrupts HRAS membrane localization without affecting its overall expression. Accordingly, pull-down assays showed that GTP-bound active HRAS levels were significantly decreased following venom exposure and were rescued by SVPLA_2_ inhibition (Fig. 3F, G).

### SVPLA_2_ disrupts lipid raft integrity and suppresses HRAS palmitoylation to block its plasma membrane localization

To elucidate the mechanism of impaired HRAS membrane localization, we focused on post-translational modification, as bioinformatic analyses indicated its prominent role in SVPLA_2_-induced nephrotoxicity. Given that palmitoylation is essential for HRAS membrane targeting, we first examined its palmitoylation status (30). Acyl–biotin exchange assays showed that HRAS palmitoylation was markedly reduced by venom exposure and significantly restored by varespladib (Fig. 3H, I). We next explored whether this reduction resulted from altered expression of known palmitoylation-modifying enzymes (30, 45). Results indicated that the major palmitoyltransferase ZDHHC9 and the depalmitoylating enzymes APT1 and APT2 showed no significant changes at the mRNA or protein level (Fig. 3J–O), However, we cannot exclude the possibility that these enzymes were regulated post-translationally or that their catalytic activities were altered by SVPLA_2_ exposure.

In parallel, lipid raft integrity is a critical prerequisite of HRAS palmitoylation and membrane compartmentalization (32, 33). Given the phospholipid-hydrolyzing activity of SVPLA_2_, we assessed lipid raft organization. Cholera toxin subunit B (Ctx-B) staining revealed pronounced disruption of lipid raft integrity in venom-treated samples, partially restored by varespladib (Fig. 3P). Disrupting lipid rafts with methyl-β-cyclodextrin (MβCD) phenocopied venom effects, reducing HRAS membrane localization, GTPase activity, and palmitoylation, whereas exogenous palmitic acid (PA) partly rescued these effects (Fig. 3R–Y). Thus, these results indicate that SVPLA_2_ impairs HRAS palmitoylation primarily by disrupting lipid raft integrity, thereby suppressing HRAS membrane localization and downstream GTPase activity.

### SVPLA_2_ suppresses PI3K–AKT signaling and promotes mitochondria-mediated apoptosis

**Fig. 4:**
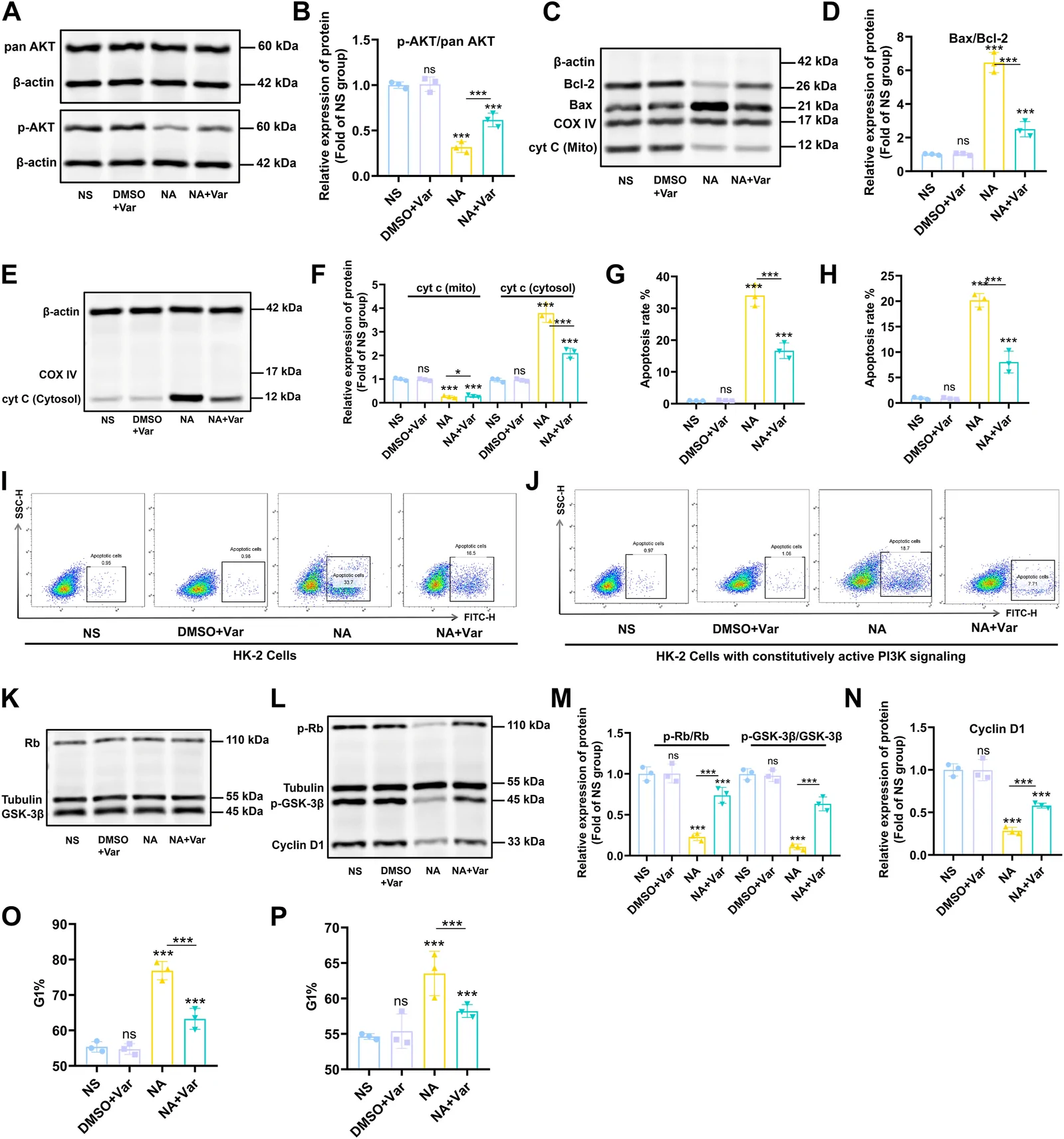
SVPLA_2_ induces PI3K-Akt inhibition-dependent mitochondrial apoptosis and G1/S cell cycle arrest. A, B: Protein levels of p-AKT relative to total AKT were determined by Western blotting and quantified. C, D: The Bax-to-Bcl-2 ratio was measured by immunoblotting and quantified. E, F: Cyt c abundance in mitochondrial and cytosolic compartments was analyzed by Western blotting and shown with quantification. G, H: Flow cytometric analysis of apoptosis following venom exposure and varespladib treatment in HK-2 cells. I, J: Apoptosis analysis with constitutively active PI3K signaling. K, L: Western Blot analysis of total and phosphorylated Rb and GSK-3β, and Cyclin D1 expression. M: Quantification of p-Rb/Rb and p-GSK-3β/GSK-3β ratios. N: Quantification of Cyclin D1 protein levels. O: Flow cytometric quantification of cell cycle distribution showing G1 phase accumulation following venom exposure. P: Cell cycle analysis with constitutively active PI3K signaling. All data are shown as mean ± SD. Significance levels: ∗*P* < 0.05, ∗∗∗*P* < 0.001. ns denotes not significant versus the specified group indicated group. n = 3 (independent biological replicates).

Based on our proteomic data and the fact that HRAS activates PI3K–AKT, we first examined whether SVPLA_2_-induced HRAS dysfunction leads to PI3K–AKT inactivation (24, 25, 46). Immunoblotting showed that venom reduced p-AKT without altering total AKT, an effect reversed by varespladib, confirming PI3K–AKT inactivation (Fig. 4A, B). Given the established link between PI3K–AKT inactivation and intrinsic apoptosis, we next assessed mitochondrial apoptotic pathways (46). SVPLA_2_ increased the Bax/Bcl-2 ratio and mitochondrial Cyt c release (Fig. 4C–F), and flow cytometry confirmed elevated apoptosis. Notably, enforced PI3K activation substantially attenuated venom-induced apoptosis (Fig. 4G–J; Supplementary Fig. S1 in Supplementary Material 1), further supporting that PI3K–AKT suppression drives mitochondrial apoptosis in this setting.

### SVPLA_2_ induces G1/S cell cycle arrest via PI3K–AKT inhibition–mediated GSK-3β activation

**Fig. 5:**
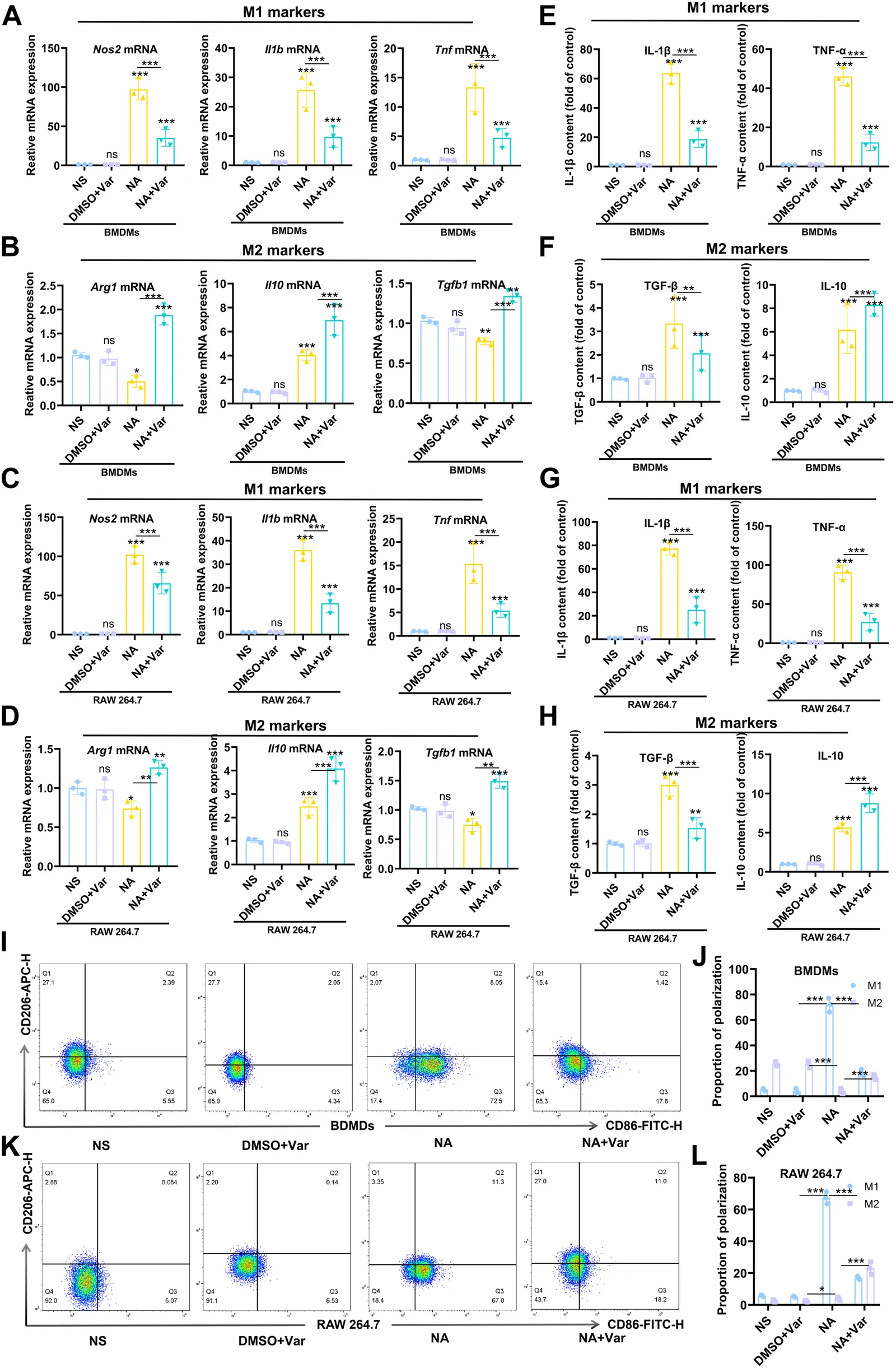
SVPLA_2_ regulates macrophage polarization and function. A–D: RT–PCR of M1-or M2-associated markers in BMDMs and RAW 264.7 cells. E–H: ELISA-based quantification of M1-and M2-associated cytokines in macrophage culture supernatants. I, J: Quantitative analysis of CD86^+^ (M1-type) and CD206^+^ (M2-type) in BMDMs. K, L: Corresponding analysis in RAW 264.7 cells. All data are shown as mean ± SD. Significance levels: ∗*P* < 0.05, ∗∗*P* < 0.01, ∗∗∗*P* < 0.001. ns denotes not significant versus the specified group indicated group. n = 3 (independent biological replicates).

Proteomic analysis also identified CCND1 as a critical responsive target and revealed enrichment of cell cycle pathways. Given that PI3K–AKT signaling regulates cell cycle progression, we examined whether its inactivation leads to cell cycle dysregulation (47, 48). Venom exposure reduced Rb phosphorylation, inhibitory GSK-3β phosphorylation, and Cyclin D1 protein levels, all restored by varespladib (Fig. 4K–N). Since GSK-3β lies downstream of the PI3K-AKT pathway and negatively regulates the stability of Cyclin D1, these results indicate that SVPLA_2_-induced PI3K–AKT suppression activates GSK-3β and results in the degradation of Cyclin D1 (49). Flow cytometry revealed reduced S-phase entry and G1-phase accumulation following venom treatment, indicating G1/S arrest (Fig. 4O; Supplementary Fig. S2 in Supplementary material 1). Similarly, enforced PI3K activation attenuated G1 accumulation and restored S-phase progression (Fig. 5P; Supplementary Fig. S2 in Supplementary material 1). Together, these findings demonstrate that SVPLA_2_-induced HRAS–PI3K–AKT disruption promotes G1/S arrest via GSK-3β activation and Cyclin D1 destabilization.

### SVPLA_2_ induces M1 polarization and impairs efferocytosis in macrophages

We previously demonstrated that *N. atra* SVPLA_2_ significantly induces M1 polarization of BMDMs, accompanied by substantial release of inflammatory cytokines (43). To determine whether the effect of SVPLA_2_ on macrophage polarization is conserved, we further performed experiments in RAW 264.7 cells and compared them with BMDMs. Notably, *N. atra* SVPLA_2_ exposure resulted in marked upregulation of both transcriptional and translational levels of M1-related markers in both cell types (Fig. 5A–H). Interestingly, certain M2-associated markers (*Il10* and *Tgfb1*) also exhibited moderate increases upon SVPLA_2_ treatment. Nevertheless, flow cytometry confirmed that SVPLA_2_ treatment significantly increased the proportion of CD86^+^ cells while decreasing the proportion of CD206^+^ cells (Fig. 5I–L), indicating that the overall polarization profile remained predominantly pro-inflammatory.

In addition, we have shown that SVPLA_2_ induces cell cycle arrest and apoptosis in renal tubular epithelial cells through inhibition of the PI3K–AKT pathway. Although apoptosis is a form of programmed cell death, persistent and massive accumulation of apoptotic cells may lead to more severe pathological consequences, highlighting the importance of timely and efficient clearance by macrophages (46). This process, termed efferocytosis, is primarily mediated by M2 macrophages (41, 43). Interestingly, we independently reproduced our previous finding that SVPLA_2_ suppresses efferocytosis in BMDMs across both macrophage cell lines (43), with flow cytometry showing that SVPLA_2_ significantly impaired apoptotic cell engulfment in both RAW 264.7 cells and BMDMs (Supplementary Fig. S3 in Supplementary Material), suggesting that SVPLA_2_ exerts essentially consistent effects on macrophage polarization and function, despite the cells originating from different sources.

### Non-targeted metabolomics reveals that SVPLA_2_ induces metabolic remodeling in BMDMs

The metabolic preference of classically activated M1 macrophages is glycolysis, whereas alternatively activated M2 macrophages preferentially metabolize fatty acids via FAO (41, 42, 43, 50). To comprehensively understand the impact of SVPLA_2_ on the metabolic profile of macrophages, we first performed non-targeted metabolomic profiling in BMDMs (n = 6). Principal component analysis distinctly separated the NS, NA, and NA + Var groups, indicating robust metabolic remodeling upon venom exposure (Supplementary Fig. S4 in Supplementary Material 1).

**Fig. 6:**
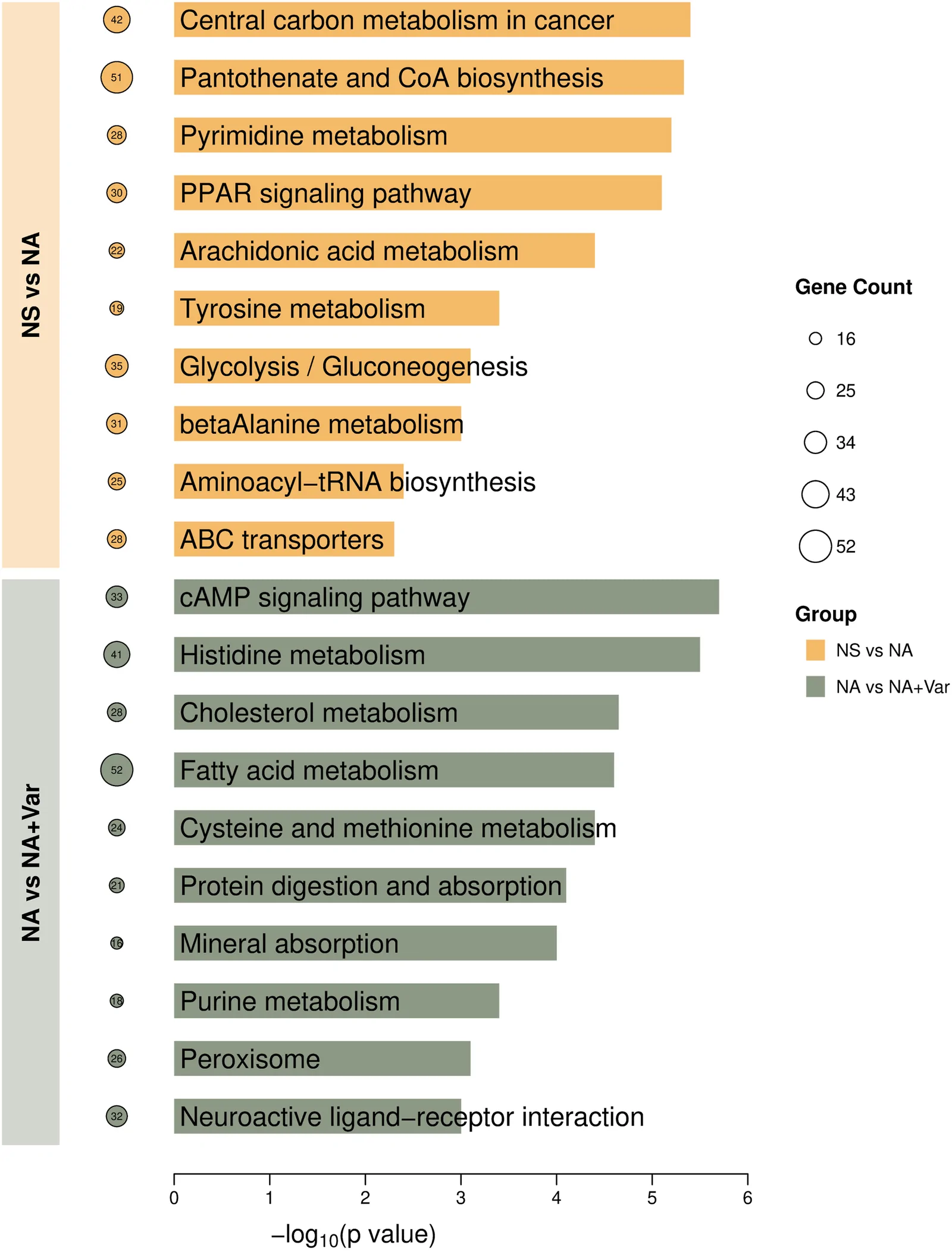
Non-targeted metabolomics-based KEGG analysis revealed that SVPLA_2_ can induce metabolic remodeling in BMDMs. n = 6 (independent biological replicates).

KEGG pathway enrichment analysis further elucidated the metabolic direction (Fig. 6). In the NS versus NA comparison, the “Glycolysis/Gluconeogenesis” pathway was significantly enriched. Additionally, “Central carbon metabolism in cancer” and “Arachidonic acid metabolism” were also enriched, further supporting the establishment of a pro-inflammatory metabolic phenotype. Notably, M2-related pathways such as “PPAR signaling pathway” and “beta-Alanine metabolism” (involved in carnitine synthesis) were also enriched, suggesting that SVPLA_2_ simultaneously perturbs fatty acid oxidation pathways. In the NA versus NA + Var comparison, “Fatty acid metabolism” and “Peroxisome” (a key site for fatty acid β-oxidation) were significantly enriched, along with the “cAMP signaling pathway”. These alterations are consistent with the partial reversal of fatty acid metabolic disturbances following varespladib intervention. Notably, the enrichment of “Histidine metabolism” corresponded to the inhibition of histamine production, further confirming the blockade of inflammatory metabolic pathways by the SVPLA_2_ inhibitor.

Collectively, the KEGG analysis supports that SVPLA_2_ induces metabolic reprogramming toward glycolysis while suppressing fatty acid oxidation, thereby driving a pro-inflammatory M1-like phenotype; varespladib intervention partially reverses these metabolic alterations.

### SVPLA_2_ drives macrophage polarization by inducing metabolic reprogramming toward glycolysis

**Fig. 7:**
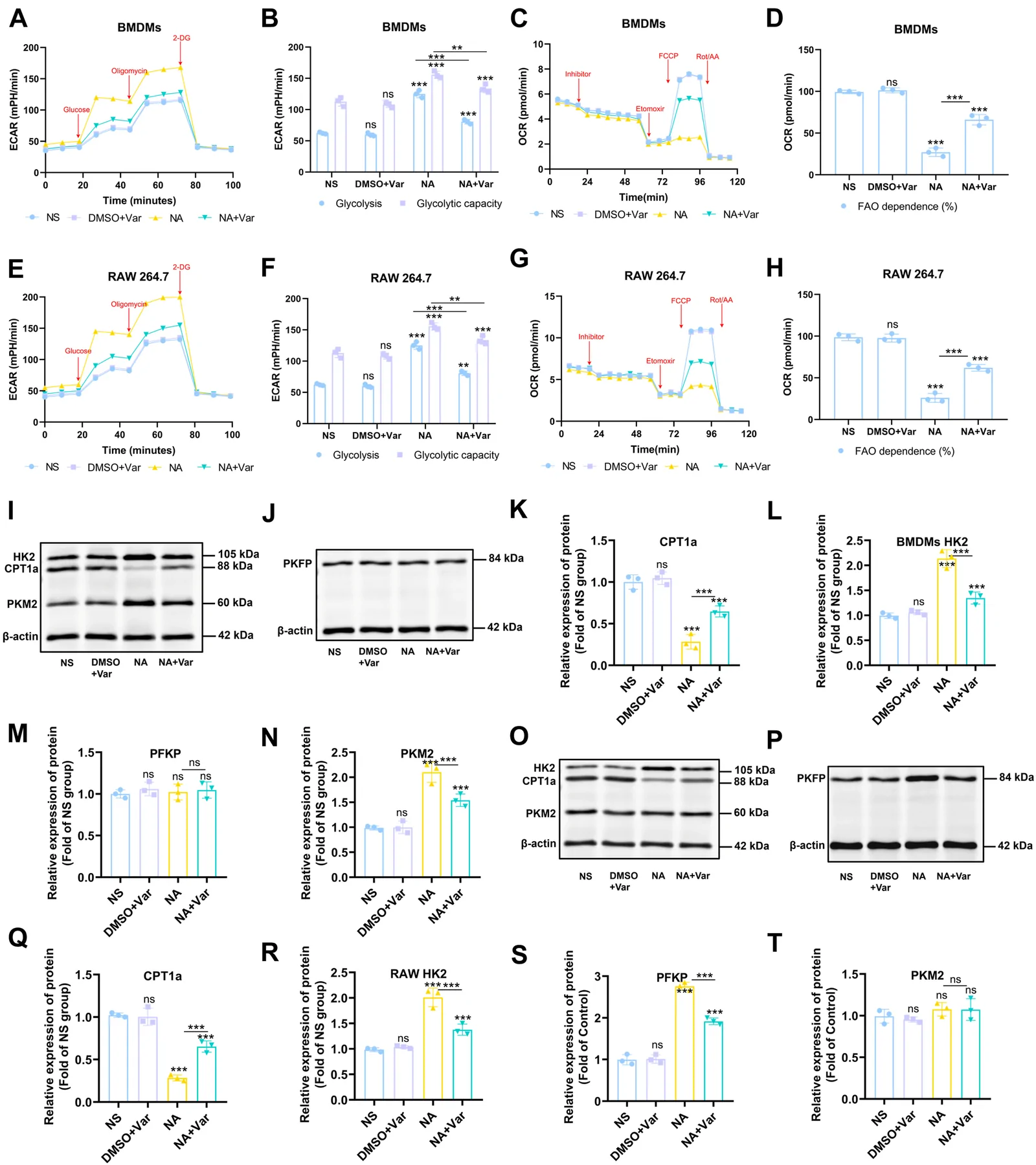
SVPLA_2_ induces metabolic reprogramming in macrophages. A–B: ECAR profiles in BMDMs. The data represent glycolysis and glycolytic capacity. C–D: FAO flux profiles in BMDMs. The inhibitor represented by etomoxir (a CPT1a inhibitor). (E–F) ECAR profiles in RAW 264.7 cells. G–H: FAO flux profiles in RAW 264.7 cells. The inhibitor represented by etomoxir (a CPT1a inhibitor). I–N: Immunoblot and quantification analysis of glycolytic and FAO enzymes in BMDMs. O–T: Immunoblot and quantification analysis of glycolytic and FAO enzymes in RAW 264.7 cells. All data are shown as mean ± SD. Significance levels: ∗∗*P* < 0.01, ∗∗∗*P* < 0.001. ns denotes not significant versus the specified group indicated group. n = 3 (independent biological replicates).

Subsequently, we assessed metabolic flux using Seahorse assays to validate the results of non-targeted metabolomics. A significant increase in ECAR was observed in BMDMs following venom exposure, pointing to elevated glycolytic activity and glycolytic capacity, while concomitantly reducing FAO dependence and FAO capacity, as assessed by oxygen consumption rate–based FAO stress tests. Similar metabolic shifts were observed in RAW 264.7 macrophages, indicating that SVPLA_2_-induced metabolic reprogramming is conserved across macrophage models (Fig. 7A–H).

Our previous studies demonstrated that SVPLA_2_ can induce upregulation of HK2 and PKM2 expression in BMDMs (43). To further elucidate the molecular mechanisms underlying this metabolic shift, we measured changes in the expression levels of key glucose metabolism enzymes in both types of macrophages. Immunoblot analysis showed marked downregulation of carnitine palmitoyltransferase IA (CPT1a), the rate-limiting enzyme for FAO, in both BMDMs and RAW 264.7 cells after SVPLA_2_ exposure, and concurrent upregulation of HK2. In contrast, the expression patterns of phosphofructokinase (PFKP) and PKM2 exhibited cell–type–specific differences between BMDMs and RAW 264.7 cells. Despite this heterogeneity at the enzyme level, glycolytic flux was consistently enhanced in both macrophage systems, indicating functional convergence toward a glycolysis-dominant metabolic state (Fig. 7I–T).

### HK knockout reverses the SVPLA_2_-induced M1 polarization

**Fig. 8:**
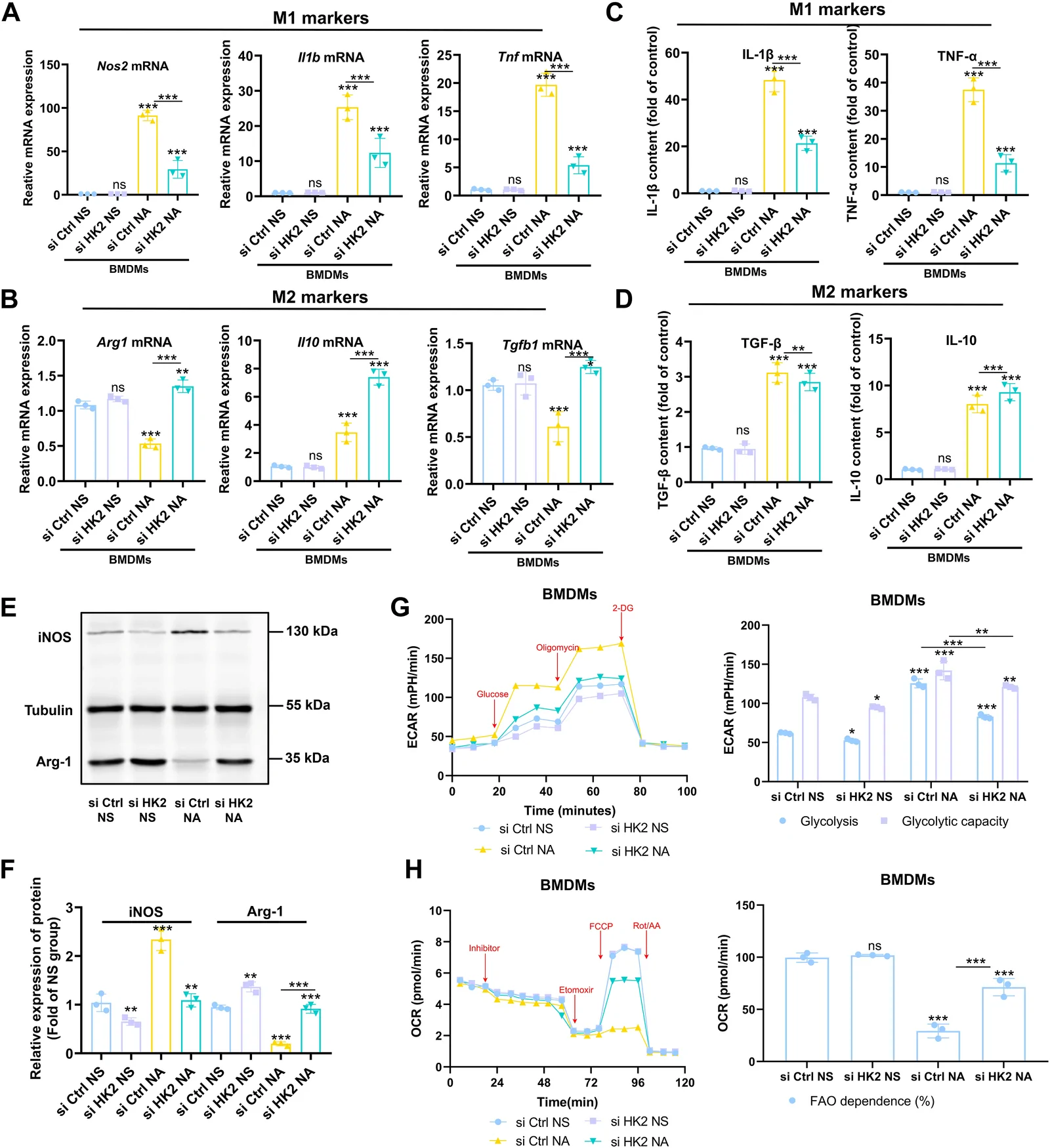
HK2 silencing attenuates SVPLA_2_-induced M1 polarization and restores macrophage metabolic homeostasis. A, B: RT–PCR of M1-or M2-associated markers in BMDMs following *Hk2* gene knockdown. C, D: ELISA-based quantification of M1-and M2-associated cytokines in macrophage culture supernatants. E: Immunoblot analysis of iNOS and Arg-1 protein expression in BMDMs after *Hk2* gene silencing. (F) Quantification of proteins expression protein levels normalized to tubulin. (G) ECAR profiles and quantification of glycolysis and glycolytic capacity in BMDMs following *Hk2* gene knockdown. (H) FAO flux profiles in BMDMs following *Hk2* gene knockdown. The inhibitor represented by etomoxir (a CPT1a inhibitor). All data are shown as mean ± SD. Significance levels: ∗∗*P* < 0.01, ∗∗∗*P* < 0.001. ns denotes not significant versus the specified group indicated group. n = 3 (independent biological replicates).

Given that HK2 protein is upregulated in both types of macrophages, we employed siRNA-mediated knockdown of *Hk2* gene in BMDMs (Supplementary Fig. S5 in Supplementary material 1). RT-PCR and ELISA analyses demonstrated that silencing HK2 expression in BMDMs markedly attenuated SVPLA_2_-driven M1 polarization while partially restoring M2 polarization (Fig. 8A–D). Consistently, immunoblotting of canonical M1 and M2 polarization markers corroborated these findings (Fig. 8E, F). Moreover, assessments of glycolytic flux and FAO flux further confirm that HK2 represents an effective metabolic intervention target for restoring macrophage homeostasis (Fig. 8G, H).

## Discussion

Globally, envenomation by Elapidae snakes frequently causes delayed and severe systemic complications (1, 2, 3), among which AKI is one of the most prevalent and clinically severe outcomes (5, 6, 7, 9). However, the current management of venom-associated AKI remains largely supportive, and mechanism-based interventions are still lacking.

Although the venom composition of *N. atra* is vary considerably depending on multiple factors (11, 12), 3FTxs and SVPLA_2_ consistently remain the predominant components of its venom proteome (11, 12, 13, 14, 15, 16). 3FTxs are widely recognized as the most abundant components. This toxin family is primarily responsible for the cytotoxic and neurotoxic activities of cobra venoms (13, 14, 15, 16, 51, 52). Although SVPLA_2_ typically constitutes a smaller fraction of the *N. atra* venom proteome, our findings demonstrate that inhibition of SVPLA_2_ with varespladib substantially attenuates venom-induced AKI, underscoring its disproportionate pathogenic contribution, consistent with previous reports (7, 8, 10, 20, 43). A growing body of evidence supports the concept of synergistic interactions among snake venom toxins, as SVPLA_2_ neutralization reduces venom lethality and pathogenicity even when SVPLA_2_ constitutes only a minor fraction of the venom proteome (21, 53). One possible mechanism is that SVPLA_2_ hydrolyzes the sn-2 ester bond of membrane phospholipids, thereby compromising membrane integrity and facilitating the cytotoxic actions of other components. These components would otherwise have limited activity against intact cellular barriers. Herein, we hypothesize that SVPLA_2_ may serve as a membrane-disrupting facilitator for 3FTxs. In this model, SVPLA_2_ enhances the access and cytotoxic actions of 3FTxs, which otherwise have limited activity against intact cellular barriers. This functional interplay would explain why inhibiting SVPLA_2_ produces a marked protective effect against AKI, despite its lower abundance relative to 3FTxs. It also accounts for the observation that varespladib only partially reverses the full spectrum of venom-induced pathology. But the synergistic interplay between SVPLA_2_ and 3FTxs remains a mechanistic hypothesis that requires further experimental validation.

Conventional antivenoms have limited efficacy against established tissue injury due to poor tissue penetration and an inability to neutralize membrane-bound toxins (54, 55). In contrast, small-molecule SVPLA_2_ inhibitors such as varespladib can rapidly access injured tissues and retain therapeutic potential after venom exposure (19, 20, 21, 22, 23). These properties highlight their promise as a mechanism-based strategy for venom-induced AKI. Nevertheless, circulating concentrations of varespladib were not monitored in the present study, which constitutes a limitation regarding its pharmacokinetic profile in our experimental model. It should be noted that we primarily used varespladib as a pharmacological tool to dissect the functional contribution of SVPLA_2_ within the complex venom milieu. Future studies incorporating serial blood sampling and comparing different dosing regimens would be valuable to more precisely define the exposure-response relationship of varespladib in this snakebite model.

HRAS is a small GTPase whose plasma membrane localization depends on palmitoylation, a modification essential for PI3K–AKT activation (24, 27, 28, 29). For instance, ZDHHC18-mediated HRAS palmitoylation promotes MEK–ERK signaling and renal fibrosis (45). Herein, SVPLA_2_ exposure disrupted HRAS membrane localization and GTPase activity without altering total HRAS protein or the mRNA/protein abundance of known palmitoylation-related enzymes, suggesting that additional regulatory mechanisms may be involved. Emerging evidence indicates that the integrity of lipid rafts represents an additional determinant of HRAS membrane localization, functioning as a structural platform for palmitoylation modification (31, 32, 33). These membrane microdomains, enriched in cholesterol, sphingolipids, and scaffolding proteins such as caveolins and flotillins, compartmentalize signaling molecules including HRAS (56, 57, 58, 59, 60). Consistent with this model, Ctx-B staining showed that SVPLA2 disrupted lipid raft integrity (loss of GM1). Moreover, MβCD, which chemically mimics raft disruption, phenocopied the defects in HRAS palmitoylation and membrane localization. These findings support a mechanism in which SVPLA_2_ impairs HRAS membrane targeting, at least in part, by disrupting lipid rafts. Interestingly, another study showed that human group V secreted phospholipases A_2_ (sPLA_2_) has been shown to associate with lipid rafts and undergo flotillin-1-dependent internalization, indicating that PLA_2_-raft interactions extend beyond mere lipid hydrolysis (61). We speculate that SVPLA_2_ may compromise raft integrity through enzymatic hydrolysis of membrane phospholipids, generating lysophospholipids and free fatty acids that destabilize ordered lipid packing, and potentially through interactions with raft-associated scaffolding proteins. However, we did not systematically examine whether SVPLA_2_ differentially affects caveolin-versus flotillin-associated raft subdomains in this study. Future studies employing super-resolution imaging or biochemical raft fractionation would be valuable to dissect the precise subdomain-specific effects of SVPLA_2_ on lipid raft architecture.

Despite documented modulation of PI3K–AKT signaling by individual venom toxins, the precise molecular triggers driving these effects have largely not been identified (62, 63). This study shows that SVPLA_2_ disrupts HRAS palmitoylation and plasma membrane localization as a proximal event leading to PI3K–AKT suppression. Consequently, HRAS inhibition reduced AKT phosphorylation, inactivated GSK-3β, and downregulated CCND1, a key G1/S regulator identified in our proteomic analysis. PI3K–AKT inactivation also promoted mitochondria-mediated apoptosis in renal epithelial cells. Notably, genetic restoration of PI3K–AKT signaling rescued SVPLA_2_-induced apoptosis and cell cycle arrest, confirming the functional centrality of this pathway in SVPLA_2_-driven renal toxicity.

A usually neglected dimension is the amplifying effect of the local immune microenvironment on organ damage (35, 36). Increasing evidence suggests that snake venom components can directly modulate macrophage polarization, but prior studies have largely focused on inflammatory activation without addressing functional consequences for tissue repair. For example, snake venoms activate macrophages via reactive species or inflammasome formation (38, 39, 64). In our previous study, despite increased macrophage infiltration, the SVPLA_2_-exposed kidneys exhibited substantial accumulation of uncleared apoptotic cells, suggesting a defect in clearance rather than impaired recruitment (43). In addition, we preliminarily demonstrated that SVPLA_2_ skewed BMDMs toward a proinflammatory M1 like phenotype while suppressing M2 related markers and the function of phagocytizing apoptotic cells. Here, we observed that this polarization shift was consistently recapitulated in both RAW 264.7 cells and BMDMs, indicating that SVPLA_2_ actively blocks the reparative polarization program and that this effect is conserved across multiple macrophage models.

Metabolic reprogramming is increasingly recognized as a pivotal determinant of macrophage polarization (41, 42, 43, 50). In our previous study, we only performed targeted metabolomics to analyze glycolysis-related metabolites. We found a marked increase in glycolytic intermediates, but a detailed investigation of SVPLA2-induced metabolic remodeling in macrophages was not conducted. Therefore, in the present study, we carried out untargeted metabolomics analysis. KEGG enrichment identified multiple pathways associated with aberrant glycolysis and fatty acid oxidation, suggesting that SVPLA_2_ may not only promote proinflammatory polarization of macrophages by upregulating glycolysis but also disturb the polarization balance by interfering with fatty acid oxidation. In both RAW 264.7 cells and BMDMs, SVPLA_2_ upregulated the glycolytic regulator HK2 and downregulated the rate-limiting enzyme of FAO, CPT1a, although PKM2 and PFKP exhibited cell type-specific differences. These distinct expression patterns likely reflect metabolic heterogeneity between immortalized and primary macrophages, yet they converge on a common feature: enhanced glycolytic dependence and impaired FAO. Functionally, genetic inhibition of HK2 restored metabolic balance, normalized polarization, and reconstituted efferocytic capacity, confirming that metabolic reprogramming is a core mechanism underlying SVPLA_2_-induced macrophage dysfunction.

An intriguing observation is that SVPLA_2_ elevated IL-10 at both mRNA and protein levels despite the overall M1-polarizing effect. This is consistent with a well-established negative feedback mechanism in innate immunity, whereby activated macrophages upregulate IL-10 as an intrinsic restraint to limit excessive tissue damage (65, 66). Notably, varespladib further increased IL-10 expression, suggesting that inhibition of SVPLA_2_ preserves the macrophage’s capacity for self-limitation by alleviating inflammatory burden (67, 68). Similarly, TGF-β exhibited a discordant pattern: SVPLA_2_ suppressed its mRNA but elevated its protein abundance. This suggests post-transcriptional regulation, as TGF-β1 is secreted in a latent form and requires extracellular activation for biological activity (69, 70). Thus, SVPLA_2_ may promote release of active TGF-β from extracellular stores without de novo transcription. In HK2-knockdown macrophages, both IL-10 and TGF-β protein were further elevated upon SVPLA_2_ treatment, suggesting that HK2-mediated glycolysis negatively regulates these immunomodulatory factors (66, 71, 72). Nevertheless, neither IL-10 elevation nor TGF-β accumulation overrode the dominant M1 polarization, as evidenced by robust upregulation of pro-inflammatory markers.

The regulatory relationship between the cGAS-STING signaling pathway and HK2 expression has been extensively reported. Chen *et al.* demonstrated that IRF3 downstream of STING activation upregulates HIF-1α, which in turn elevates HK2 expression and glycolysis; glycolysis-derived ATP further reinforces STING signaling and interferon responses (73). More recently, STING was shown to stabilize HIF-1α, enhance glycolysis, and promote histone lactylation at the HK2 locus, facilitating IRF3 binding to the HK2 promoter and sustaining glycolytic flux (74). Since the canonical activation of the cGAS-STING pathway relies on the cytosolic accumulation of aberrant dsDNA, with mitochondrial DNA (mtDNA) representing one of the most frequent activating signals (75, 76), it is noteworthy that SVPLA_2_ has been reported to induce oxidative stress by targeting nuclear factor erythroid 2-related factor 2 (Nrf2), resulting in marked mitochondrial dysfunction and excessive mitophagy (20). Similarly, we also observed that SVPLA_2_ triggered mitochondrial dysfunction in HK-2 cells in the present study. Although the direct link between SVPLA_2_ and cGAS-STING activation was beyond the scope of our initial investigation, our recent work has revealed this upstream mechanism (77). Specifically, SVPLA_2_ induced mitochondrial dysfunction in macrophages, leading to mtDNA efflux and activation of the cGAS-STING pathway, which in turn upregulated HK2 expression and glycolytic activity, ultimately driving macrophages toward the M1 proinflammatory phenotype.

**Fig. 9:**
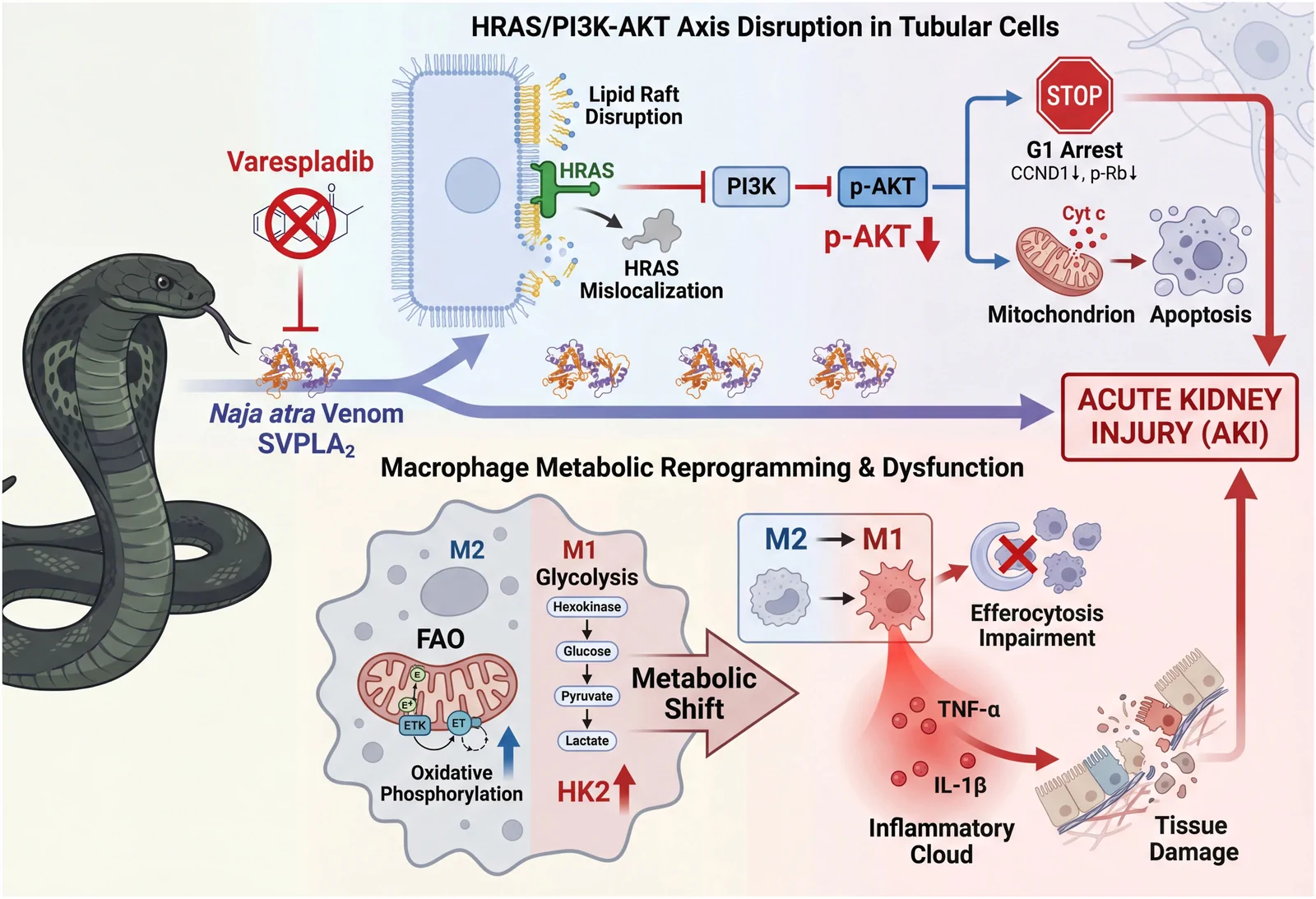
Mechanistic pathways of this study.

In addition to the limitations mentioned above, several other shortcomings of this study warrant further discussion. First, instead of isolating the SVPLA_2_ enzyme, we used whole *N. atra* venom combined with the specific SVPLA_2_ inhibitor varespladib to investigate its toxic mechanisms. This approach was chosen because snake venom is a highly complex mixture in nature, and its components do not act independently but rather synergistically. Thus, this method more accurately reflects venom complexity and allows us to assess the toxic effects and mechanisms of SVPLA_2_ within the context of whole venom. Although many previous studies have been conducted in this manner (8, 10, 20, 21, 43, 53), future studies should perform comparative analyses to identify more suitable toxicological approaches. Second, our study was largely framed within the M1/M2 paradigm, whereas macrophages exhibit substantial heterogeneity. Future studies should combine flow cytometry with ex vivo analysis of diverse macrophage subsets to more comprehensively delineate the interaction between snake venom and the innate immune network. Finally, our non-targeted metabolomics analysis in macrophages also revealed SVPLA_2_-induced alterations in pathways related to fatty acid metabolism and arachidonic acid metabolism, in addition to the glycolysis-related pathways that were the primary focus of this study. The downregulation of CPT1a further supports a metabolic shift away from oxidative metabolism in SVPLA_2_-treated macrophages. These observations raise the possibility that SVPLA_2_ may orchestrate a broader perturbation of lipid metabolism beyond glycolysis. Therefore, future studies employing lipidomics to profile bioactive lipid mediators would be valuable to comprehensively elucidate the impact of SVPLA_2_ on macrophage lipid metabolic reprogramming. Figure 9 shows the overall mechanism diagram of *N. atra* SVPLA_2_-induced AKI.

## Conclusion

This study demonstrates that varespladib exerts significant protective effects against *N. atra* venom-induced AKI. Through pharmacological inhibition of SVPLA_2_ activity, we identify SVPLA_2_ as a critical driver of the pathogenic mechanisms observed in this model. Mechanistically, SVPLA_2_ disrupts lipid raft integrity, thereby impairing HRAS palmitoylation-dependent plasma membrane localization and GTPase activity, suppressing PI3K–AKT signaling, and further promoting apoptosis and cell cycle arrest. In parallel, SVPLA_2_ reprograms macrophage metabolism, skews polarization toward a pro-inflammatory phenotype, and impairs efferocytosis. These epithelial and immune perturbations act in concert to exacerbate renal injury and hinder tissue repair. Taken together, this work extends the conventional view of snake venom toxicity by integrating analyses of direct tubular cytotoxicity and alterations in the renal immune microenvironment. It provides a mechanistic rationale for targeting SVPLA_2_-driven membrane signaling and immune reprogramming as a therapeutic strategy for venom-induced AKI.

## Data availability

All data are available from the corresponding author on reasonable request.

## Supplemental data

This article contains supplemental data.

## Conflict of interest

The authors declare that they have no conflicts of interest with the contents of this article.
