# Supplementary material for: Naja atra SVPLA2 contributes to venom-induced AKI via HRAS palmitoylation-dependent PI3K-AKT inactivation
Source: J Lipid Res. 2026 Aug 14;67(9):101123. doi: 10.1016/j.jlr.2026.101123 (PMC13579411; doi:10.1016/j.jlr.2026.101123)

Detailed materials and methods

- **Main reagents and antibodies**

Detailed information on the main reagents and antibodies are provided in Table S1 and S2.

Table S1. Main reagents.

Item Name	Source	Cat No.
Lyophilized *Naja atra* venom	Chengfeng Snake Farm	N/A
Varespladib (LY315920)	MedChemExpress	HY-13402
Methyl-β-cyclodextrin	MedChemExpress	HY-101461
Palmitic acid	MedChemExpress	HY-N0830
Cell Counting Kit 8 (CCK-8)	Abcam	ab228554
SCr Assay Kit	Nanjing Jiancheng Bioengineering Institute	C011-2-1
BUN Assay Kit	Nanjing Jiancheng Bioengineering Institute	C013-2-1
Uric Acid Assay Kit	Nanjing Jiancheng Bioengineering Institute	C012-1-1
Urine Protein Assay Kit	Nanjing Jiancheng Bioengineering Institute	C035-2-1
Myoglobin Assay Kit	Nanjing Jiancheng Bioengineering Institute	H150-1-1
Free Hemoglobin Assay Kit	Nanjing Jiancheng Bioengineering Institute	A071-1-1
Mouse IL-10 ELISA Kit	Thermo Fisher Scientific	BMS614-10
Mouse TGF-β ELISA Kit	Thermo Fisher Scientific	88-8350-88
Mouse TNF-α ELISA Kit	Thermo Fisher Scientific	BMS607-3
Mouse IL-1β ELISA Kit	Abcam	ab197742
Mem-PER™ Plus Membrane Protein Extraction Kit	Thermo Fisher Scientific	#89842
Cell Mitochondrial Isolation Kit	Beyotime	C3601
Active Ras Detection Kit	Cell Signaling Technology	#8821
Alexa Fluor 488-conjugated cholera toxin subunit B	Thermo Fisher Scientific	C34775
Seahorse XF Glycolytic Rate Assay Kit	Agilent	#103020-100
Seahorse XF Long Chain Fatty Acid Oxidation Stress Test Kit	Agilent	#103672-100

Table S2. Antibodies used in western blotting (WB), Immunohistochemistry (IHC) and flow cytometry (FC).

Application	Primary Antibodies	Dilution (v/v）	Source Species	Company	Cat No.
Primary antibodies					
WB	β-actin	1:5000	Rabbit	Abcam	ab8226
WB	Tubulin	1:5000	Rabbit	Abcam	ab7291
WB	Na^+/K^+ ATPase	1:10000	Rabbit	Proteintech Group	14418-1-AP
WB	HRAS	1:5000	Rabbit	Abcam	ab32417
WB	ZDHHC9	1:800	Rabbit	Abcam	ab217757
WB	APT1	1:2500	Rabbit	Proteintech Group	16055-1-AP
WB	APT2	1:1000	Rabbit	Abcam	ab151578
WB	AKT	1:1000	Rabbit	Cell Signaling Technology	#9272
WB	p-AKT	1:1000	Rabbit	Cell Signaling Technology	#9271
WB	Bcl-2	1:1000	Rabbit	Abcam	ab194583
WB	Bax	1:1000	Rabbit	Abcam	ab182733
WB	cyt C	1:1000	Rabbit	Cell Signaling Technology	#11940
WB	COX IV	1:1000	Rabbit	Cell Signaling Technology	#4844
WB	Rb	1:1000	Rabbit	Abcam	ab181616
WB	p-Rb	1:1000	Rabbit	Cell Signaling Technology	#8516
WB	GSK-3β	1:500	Mouse	Abcam	ab93926
WB	p-GSK-3β	1:1000	Rabbit	Cell Signaling Technology	#5558
WB	Cyclin D1	1:1000	Rabbit	Cell Signaling Technology	#2922
WB	F4/80	1:1000	Rabbit	Abcam	ab300421
WB	CD68	1:1000	Rabbit	Abcam	ab283654
WB	CPT1a	1:1000	Rabbit	Cell Signaling Technology	#12252
WB	HK2	1:10000	Rabbit	Abcam	ab227198
WB	PFKP	1:500	Mouse	Abcam	ab119796

WB	PKM2	1:10000	Rabbit	Abcam	ab150377
WB	iNOS	1:1000	Rabbit	Abcam	ab178945
WB	Arg-1	1:5000	Rabbit	Abcam	ab96183
IHC	NGAL	1:200	Rabbit	Abcam	ab125075
IHC	KIM-1	1:250	Rabbit	Abcam	ab316854
FC	F4/80	1:100	Rat	Abcam	ab6640
FC	CD86	1:400	Rat	Cell Signaling Technology	#99879
FC	CD206	1:20	Mouse	Cell Signaling Technology	#69027

Genera	Dilution (v/v）	Company	Cat No.
Secondary antibodies			
HRP Goat Anti-Rabbit IgG (for WB)	1:5000	Abcam	ab6721
HRP Goat Anti-Rabbit IgG (for IHC)	1:400	Abcam	ab6721
HRP Goat Anti-Mouse IgG (for WB)	1:5000	Abcam	ab6789

- **Cell culture and mycoplasma monitoring**

Human renal cortex proximal tubule epithelial cells (HK-2) (RRID:CVCL_0302) and murine macrophage-like cells (RAW 264.7) (RRID: CVCL_0493) (Immocell Biotechnology Co., Ltd., Xiamen, China. Cat. NO. IM-H060; IM-M028, respectively) were maintained in Dulbecco's modified Eagle's medium (DMEM) supplemented with 10% fetal bovine serum (FBS) and 1% penicillin-streptomycin at 37 °C/5% CO_2.

To ensure data rigor and reproducibility, all cell lines were maintained at a low passage number (below 10) to prevent potential genetic alterations. Mycoplasma contamination was routinely monitored in this study. Specifically, tests were performed at each passage and during every freeze-thaw cycle, all of which were negative.

- **Isolation and differentiation of BMDMs**

Bone marrow cells were harvested from the femurs and tibiae of C57BL/6J mice (6–7 weeks old, 20 ± 3 g) by flushing the marrow cavities with PBS. Cell suspensions were centrifuged at 500 × g for 5 min at 4 °C, followed by erythrocyte lysis with ACK lysis buffer. After termination of lysis with complete medium and a second centrifugation, cells were filtered through a 70-μm cell strainer to

remove bone debris and cell aggregates. Isolated bone marrow cells were seeded in culture dishes at a density of 5×10^6 cells per dish and differentiated in complete DMEM medium/12% FBS, and 25 ng/mL recombinant mouse macrophage colony-stimulating factor. On day 3, replace the culture medium; on day 5, perform a second medium replacement, and continue cell culture for a total of 7 days to ensure differentiation into BMDMs.

- **Construction of gene knockdown or overexpression cell models**

siRNA-mediated knockdown of *Hk2* in BMDMs: siRNA targeting *Hk2* and a non-targeting control siRNA were chemically synthesized by an enterprise (Biosyntech, China). The siRNA sequences were: forward (5'-3') GGCUUGAGCAGGCUAAACU; reverse (5'-3'), AGUUUAGCCUGCUCAAGCC. A scrambled siRNA was used as negative control. BMDMs were transfected with the indicated siRNAs using Lipofectamine 3000. Briefly, BMDMs were seeded to reach approximately 50% confluence at the time of transfection. siRNA were diluted in Opti-MEM and incubated with the transfection reagent to form siRNA–lipid complexes prior to addition to the cells. The final concentration of siRNA was 50 nM. Cells were harvested 24 h after transfection, and knockdown efficiency was evaluated by immunoblotting and quantitative real-time PCR.

Construction of HK-2 stable cells with constitutively active PI3K signaling: the H1047R mutation, located in the kinase domain of *PIK3CA* (*p110α*), is a well-characterized hotspot mutation known to confer constitutive PI3K activity and is widely used to model PI3K–AKT pathway activation. An oncogenic activating mutant of *p110α*, *PIK3CA^H1047R*, was stably expressed in HK-2 cells using a lentiviral expression system. The PIK3CA mutant coding sequence was cloned into a lentiviral transfer vector driven by a constitutive promoter (*EF1α*) and carrying an antibiotic resistance cassette (Puromycin). Lentiviral particles were produced using standard packaging plasmids and used to transduce HK-2 cells, followed by antibiotic selection to obtain stable polyclonal cell populations. Stable expression was confirmed by immunoblotting. Construct information is provided in Table S3.

Table S3. Sequence information.

Gene Name	Domain	Forward Primer for H1047R mutation in *p110α-*	Reverse Primer for H1047R mutation in *p110α-*
PIK3CA (p110α)	Kinase domain (Exon 20)	CAAATGAATGATGCACgTCATGGTG GCTGGACAACAAAAATGG	TGCATCATTCATTTGTTTCATGAAA TACTCCAAAGC

- **Cell viability assay**

Cell viability was assessed using the Cell Counting Kit-8 (CCK-8). For HK-2 cells, to determine the IC_{50} of *N. atra* venom, cells were treated with a concentration series ranging from 20 to 200 μM (in 20 μM increments). The protective effect of varespladib was subsequently assessed at the venom IC_{50} concentration, using varespladib gradients from 20 to 200 μg/mL (also in 20 μg/mL increments). For RAW 264.7 cells and BMDMs, cells were treated with increasing concentrations of *N. atra* venom (0, 0.5, 1, 2, 4, 8, and 10 μg/mL). Based on the experimental objective of this study, a venom concentration that maintained 80–90% cell viability while robustly inducing M1 macrophage polarization was selected as the working concentration. To investigate the dose of varespladib in macrophages, after establishing the venom working concentration, cells were treated with varespladib at graded concentrations (0, 0.3, 1, 3, and 10 μM). Cells were co-treated with varespladib and venom simultaneously for 24 h. A varespladib concentration that maintained 85–95% cell viability while significantly attenuating venom-induced M1 polarization was selected as the optimal intervention dose.

Finally, the working concentrations of *N. atra* venom were determined as 87 μg/mL for HK-2 cells, 5 μg/mL for RAW 264.7 cells, and 4 μg/mL for BMDMs. The corresponding protective concentrations of varespladib were established at 55 μM for HK-2 cells, 3.8 μM for RAW 264.7 cells, and 3 μM for BMDMs.

- **Histopathology**

Tissues were fixed in 4% paraformaldehyde for at least 48 h, embedded in paraffin, and sectioned at a thickness of 5 μm. Then, sections were deparaffinized, rehydrated, and stained with Hematoxylin and Eosin (H&E) to examine general morphology and pathological alterations, or with Periodic Acid–Schiff (PAS) to evaluate glycogen content and the integrity of the basement membrane. Images were acquired using a bright-field microscope (Nikon).

- **IHC**

Tissue sections were subjected to antigen retrieval and endogenous peroxidase blocking, followed by blocking with 10% Lowlenthal serum for 1 h. Sections were incubated with primary antibodies at 4 °C overnight and HRP-conjugated secondary antibodies at room temperature for 1 h. Signals were visualized using a DAB substrate kit, and nuclei were counterstained with hematoxylin. Images were acquired using a bright-field microscope (Nikon).

- **Assessment of renal function biomarkers**

Serum was obtained by centrifugation (2,500 × g, 4°C, 10 min). Serum levels of creatinine (SCr), blood urea nitrogen (BUN), uric acid (UA), myoglobin, and free hemoglobin were measured using Assay Kits. All measurements were performed in duplicate using a microplate reader (SpectraMax® iD3, Molecular Devices).

- **ELISA**

IL-10, TGF-β, TNF-α, IL-6 and IL-1β levels in cell culture supernatant or tissues were quantified using ELISA kits in accordance with the manufacturer's instructions. Absorbance readings at 450 nm were obtained using a microplate reader, and cytokine concentrations were interpolated from a standard calibration curve.

- **RT-qPCR**

Total RNA was isolated using TRIzol, reverse-transcribed, and amplified with SYBR Green on the Bio-Rad CFX Connect system. *Actb*-normalized expression was calculated via $2^{-\Delta\Delta Ct}$. All primers are listed in Table S4.

Table S4. Primer sequences used for RT-PCR.

Gene Name	Forward Primer Sequence (5' to 3')	Reverse Primer Sequence (5' to 3')
Hras	TCGCACTGTTGAGTCTCGGCAG	TATGCTGCCGAATCTCACGGAC
Lypla1	CTCACCACACAGCAGAAACTGG	TCCATGGCACTGGAGAACGGAA
Lypla2	CCTCTATACAGCACTTACCTGCC	CAGGTCCTTGGCACTGCCATTG
Zdhhc9	CTGCTGTGAAGTGCTTTGTGGC	TCTGTGGCAACAGGCTACTGCT
Ccnd1	GCAGAAGGAGATTGTGCCATCC	AGGAAGCGGTCCAGGTAGTTCA
Adgre1	CGTGTTGTTGGTGGCACTGTGA	CCACATCAGTGTTCCAGGAGAC
Cd68	GGCGGTGGAATACAATGTGTCC	AGCAGGTCAAGGTGAACAGCTG
Nos2	CTGCAAGAGACTTCCATCCAG	ACATCGACCCGTCCACAGTAT
Il1b	TCGGTAACTGACTTGAATGTCCA	CAGCCAGATGCAATCAATGCC
Tnf	CAGGCGGTGCCTATGTCTC	CTCCAAGCCAAAGTCCTTAGAG
Arg1	AAGGCCAACCGTGAAAAGAT	TCGCTTCCCTGTTTTAGCTGC
Il10	CGGGAAGACAATAACTGCACCC	CGGTTAGCAGTATGTTGTCCAGC
Tgfb1	TGATACGCCTGAGTGGCTGTCT	CACAAGAGCAGTGAGCGCTGAA

Actb	GGCTGTATTCCCCTCCATCG	CCAGTTGGTAACAATGCCATGT

- **Plasma membrane protein extraction**

Plasma membrane proteins were isolated from HK-2 cells using a commercial extraction kit per the manufacturer's protocol. Briefly, harvested cells were washed, resuspended in a hypotonic extraction buffer, and incubated at 4°C for 30 min with gentle agitation. Following low-speed centrifugation, the supernatant was further incubated and subsequently centrifuged to induce phase separation. The enriched plasma membrane fraction was collected from the lower phase.

- **Mitochondrial fractionation**

Mitochondrial and cytosolic fractions were isolated using a commercial Mitochondria Isolation Kit according to the manufacturer's instructions. Briefly, harvested cells were homogenized in a provided isotonic buffer. The homogenates were then subjected to sequential differential centrifugation: an initial low-speed step (600 × g, 4°C, 10 min) to remove nuclei and unbroken cells, followed by a high-speed centrifugation (12,000 × g, 4°C, 20 min) to pellet the mitochondrial fraction. The resulting supernatant was collected as the cytosolic fraction. To confirm the success of fractionation, the efficacy of this isolation procedure was verified by WB analysis using dual internal controls: COX IV (mitochondrial marker) and β-actin (cytosolic marker).

- **Immunoblotting**

Proteins were separated by SDS-PAGE, transferred to PVDF membranes, blocked (5% BSA, 2 h), and probed with primary (4 °C, overnight) and HRP-secondary antibodies (1 h). The signal was visualized using enhanced chemiluminescence reagents and documented using an imaging system.

- **Active HRAS pull-down assay**

The levels of GTP-bound active HRAS were determined using the Active RAS Pull-down and detection kit. Briefly, cells were lysed in the provided buffer, and clarified lysates were incubated with Raf1-RBD agarose beads to specifically pull down GTP-RAS. After extensive washing, the bound proteins were subjected to WB analysis using HRAS-specific antibodies. Input lysates and GppNHp (Positive control)/GDP (Negative control) -loaded samples were included as controls.

- **HRAS palmitoylation detection**

Palmitoylation of HRAS was assessed using the acyl-biotin exchange (ABE) method. Cells were

lysed in a buffer containing 1% SDS, and free thiols were blocked by incubating with 50 mM N-ethylmaleimide (NEM) for 2 h at 4°C. Excess NEM was removed by acetone precipitation (three volumes of cold acetone, –20°C for 1 h). The protein pellet was resuspended in lysis buffer, divided equally into two tubes, and incubated with or without 1 M hydroxylamine (HAM, pH 7.2) for 2 h at room temperature to specifically cleave thioester-linked palmitoyl groups. The newly exposed thiols were labeled with 0.5 mM HPDP-biotin (biotin-HPDP) for 1 h at room temperature. Biotinylated proteins were captured using streptavidin-agarose beads for 2 h at 4°C, followed by five washes with lysis buffer. The precipitated proteins were eluted by boiling in SDS-PAGE loading buffer and subjected to immunoblotting with an anti-HRAS antibody. Samples treated without hydroxylamine served as negative controls. Notably, parallel control experiments confirmed equal total HRAS protein levels across all groups as described above, allowing direct comparison of palmitoylation signals between groups.

- **Lipid raft staining**

Lipid rafts were visualized in cells using Alexa Fluor 488-conjugated cholera toxin subunit B (Ctx-B), which binds to the raft-associated ganglioside GM1. After washing with ice-cold PBS, cells were labeled with Alexa Fluor 488-conjugated Ctx-B. Images were acquired via LSM 910 confocal microscopy (ZEISS).

- **FC analysis of apoptosis**

Apoptosis was detected using a FITC-conjugated TUNEL assay kit. Briefly, after experimental treatments, cells were harvested, fixed, permeabilized, and incubated with the TUNEL reaction mixture. Following washing, cells were resuspended in PBS and analyzed immediately. Apoptotic cells (FITC-positive) were distinguished from viable cells (FITC-negative) based on fluorescence intensity using flow cytometer. All flow cytometric analysis in this study was performed on a BD LSRFortessa X-20 instrument (BD Biosciences).

- **FC analysis of cell cycle distribution**

Treated cells were harvested, fixed in 70% ice-cold ethanol overnight at -20°C, washed, and then stained with propidium iodide (PI) solution containing RNase A. After incubation in the dark at room temperature for 30 min, the DNA content of the cells was analyzed by flow cytometry. The percentages of cells in G0/G1, S, and G2/M phases were determined, with doublets excluded by gating on the area vs. width signal of PI fluorescence.

- **Efferocytosis assay**

Apoptotic HK-2 cells were induced by ultraviolet irradiation and subsequently labeled with 5 μM carboxyfluorescein succinimidyl ester (CFSE). RAW 264.7 cells or BMDMs were then co-cultured with the CFSE-labeled apoptotic cells at a 5:1 ratio for 30 min at 37°C. Non-internalized apoptotic cells were removed by gentle washing. Macrophages were detached, fixed, and immunostained with a phycoerythrin (PE)-conjugated anti-F4/80 antibody. The percentage of F4/80-positive macrophages that had engulfed apoptotic cells (CFSE-positive) was determined by flow cytometry.

- **FC analysis of macrophage polarization**

Macrophage polarization was assessed by surface marker staining. Following treatments, RAW 264.7 cells or BMDMs were harvested, washed, and stained with fluorescently conjugated antibodies against specific markers: fluorescein isothiocyanate (FITC)-anti-CD86 (for M1 phenotype) and allophycocyanin (APC)-anti-CD206 (for M2 phenotype). Corresponding isotype controls were used for gating. After incubation and washing, cells were analyzed using flow cytometer. The polarization state was determined by the median fluorescence intensity (MFI) and the percentage of positive cells for each marker.

- **Glycolytic flux measurement**

The glycolytic function of macrophages was assessed by measuring the extracellular acidification rate (ECAR) using a Seahorse XFe24 Analyzer. Briefly, cells were seeded into XF24 cell culture microplates and incubated overnight. Prior to the assay, the culture medium was replaced with XF DMEM medium (pH 7.4) and incubated in a non-CO_2 incubator at 37°C for 1 h. The following compounds were injected sequentially during the assay: 10 mM glucose, 1 μM oligomycin, and 50 mM 2-deoxy-D-glucose (2-DG).

- **FAO flux measurement**

FAO flux was specifically assessed by measuring the long-chain FAO stress using a Seahorse XFe24 Analyzer. Cells were seeded in XF24 plates and treated as required. Prior to assay, cells were equilibrated for 60 min at 37°C in substrate-limited medium (Seahorse XF Base Medium supplemented with 1.0 mM L-carnitine, without glucose, glutamine, or pyruvate). During the assay, the following compounds were sequentially injected: 100 μM palmitate-BSA conjugate or BSA vehicle control, 40 μM etomoxir (CPT1a inhibitor), 1.5 μM FCCP and a mixture of rotenone (2 μM) & antimycin A (2 μM).

- **Quantitative proteomic analysis**

Protein samples from kidney were quantified (n = 6), and equal amounts underwent reduction, alkylation, and trypsin digestion. The digested peptides were desalted, labeled using TMTpro 16-plex reagents, and quenched. Labeled samples were pooled at a 1:1 ratio, desalted again, and fractionated via high-pH reverse-phase HPLC. LC-MS/MS analysis was performed on a Q-Exactive HF-X mass spectrometer coupled with an EASY-nLC 1200 liquid chromatography system. Peptide separation was achieved on a C18 column using a 120-min acetonitrile gradient. The mass spectrometer operated in data-dependent acquisition mode, acquiring full MS scans at a resolution of 120,000 followed by higher-energy collisional dissociation (HCD) fragmentation of the top 20 precursors.

Raw mass spectrometric data were processed using Proteome Discoverer (version 2.4). Database searching was carried out with Mascot (version 2.6) against the UniProt mouse reference proteome. Search parameters allowed up to two missed tryptic cleavage sites, specified TMTpro tags and carbamidomethylation as fixed modifications, and methionine oxidation as a variable modification. For protein quantification, the intensities of TMTpro reporter ions were extracted from MS/MS spectra via the Reporter Ions Quantifier node within Proteome Discoverer. To correct for variability in sample loading, reporter ion intensities were normalized to the total peptide intensity measured in each channel. Only proteins identified by at least two unique peptides were considered quantifiable. Peptide and protein identifications were filtered to a false discovery rate (FDR) of 1%.

For differential expression analysis, proteins were quantified and statistical significance was assessed using Student's t-test between groups. To correct for multiple hypothesis testing, the Benjamini-Hochberg (BH) method was applied to control the false discovery rate (FDR) at a threshold of $q < 0.05$. Proteins with $|\log_2 \text{fold change}| \geq 1.5$ and $q < 0.05$ were considered differentially expressed proteins (DEPs). Downstream bioinformatic analyses, including heatmap generation, GO enrichment, KEGG pathway enrichment, and COG analysis were conducted using R 3.4. To explore potential toxic targets and mechanisms, DEPs were subjected to dual-algorithm analysis (MCODE and cytoHubba) within Cytoscape.

- **Metabolomics analysis**

For untargeted metabolomic profiling, metabolites were extracted from treated BMDMs from the three groups (n = 6 per group). The analysis was conducted by a commercial provider (Oebiotech, Shanghai, China). Cell pellets were homogenized in 80% methanol containing 250 ng/μL

$^{13}C_6$-phenylalanine as an internal standard. After centrifugation at 12,000 × g for 10 min at 4°C, the supernatants were dried under nitrogen and stored at −80°C until analysis. Prior to LC-MS analysis, residues were reconstituted in mobile phase. A pooled quality control (QC) sample was prepared by mixing equal aliquots of all samples and injected periodically throughout the analytical run to monitor system stability and reproducibility.

Chromatographic separation was performed on a Waters ACQUITY UPLC system equipped with a BEH C18 column (2.1 × 100 mm, 1.7 μm). The mobile phases consisted of 0.1% formic acid in water (A) and 0.1% formic acid in acetonitrile (B), using a gradient elution program. The gradient started at 2% B, increased to 50% B over 15 min, then to 95% B over 2 min, held for 3 min, and re-equilibrated at 2% B for 5 min. The flow rate was 0.3 mL/min, and the column temperature was maintained at 40°C. Mass spectrometry was carried out on a Q-Exactive Orbitrap mass spectrometer in both positive and negative electrospray ionization modes. The full scan range was m/z 100–1200 at a resolution of 70,000, and MS/MS data were acquired in data-dependent mode at a resolution of 17,500.

Raw data were processed with Compound Discoverer 3.2 for peak detection, alignment, and filtering. Metabolites were putatively identified by matching accurate mass (≤ 5 ppm), retention time, and MS/MS spectra against the Human Metabolome Database (HMDB), KEGG, and an in-house library. Data were normalized to median intensity per sample and $\log_2$-transformed. Differential metabolites between groups (NS vs. NA and NA vs. NA+Var) were selected based on orthogonal partial least-squares discriminant analysis (OPLS-DA) with variable importance in projection (VIP) > 1, $|\log_2$ fold change$| > 1$, and Student's t-test $p < 0.05$. For functional interpretation, KEGG pathway enrichment analysis was conducted using the clusterProfiler package in R. A hypergeometric test was applied, and p-values were adjusted by the Benjamini-Hochberg false discovery rate (FDR) method. Pathways with FDR < 0.05 were considered significantly enriched. The top 10 enriched pathways for each comparison were presented.

Table S5. Mcode and cytoHubba algorithms identified intersecting differentially expressed proteins.

Group	Algorithm	Gene Name
NS *vs*. NA	Mcode	*Akr1b8, Aktip, Aldh1a2, Asxl2, Car1, Car14, Ccnd1, Ceacam1, Cebpz, Cfd, Chmp3, Ergic1, Ethe1, Exosc2, Fdx1, Fgd4, Ftcd, Gbp7, Gcc2, Ghitm, Golga7, Gpld1, Hras, Hsdl2, Irak1, Itga1, Itgb3, Kng1, Rab1b, Rab27b, Rae1, Rpa1, Sdsl, Sec61b, Slc5a10, Snca, Sumo1, TUbe2a, Ube2m, Ubtf*
	cytoHubba	*Abcc2, Acaa2, Ccnd1, Ccs, Cd5l, Dctn3, Ddx54, Hras, Hsdl2, Irak1, Isg15, Paip1, Pak1, Pcbp1*
NA *vs*. NA+Var	Mcode	*Blvra, Blvrb, C1qa, C2, C3, Cab39, Car1, Car14, Ccnd1, Ccs, Cd5l, Ccnd1, Cdc16, Eif3g, Epb42, Ergic1, Ethe1, Exoc6b, Exosc2, Fdx1, Fgd4, Ftcd, Gbp7, Gcc2, Ghitm, Golga7, Gpld1, Hnrnpk, Hras, Hsdl2, Irak1, Isg15, Isg20, Itga1, Itgb3, Kng1, Ldah*
	cytoHubba	*Acnat1, Apoa5, Btf3, Ccnd1, Cct2, Col6a1, Cyp27a1, Eny2, Farsa, Gimap1, Hras, Sar1a*

Fig. S1. Characterization of HK-2 cells with constitutive activation of the PI3K–AKT signaling pathway.

(A) Western blot analysis of p-AKT and total AKT expression in HK-2 cells transfected with vector control or PIK3CA^H1047R^.

(B) Quantitative analysis of the p-AKT/total AKT. Data are expressed as fold change relative to the vector control group.

All data are shown as mean ± SD. Significance levels: * $p < 0.05$, ** $p < 0.01$, and *** $p < 0.001$. ns denotes not significant versus the specified group indicated group. n = 3 biological replicates (in vitro).

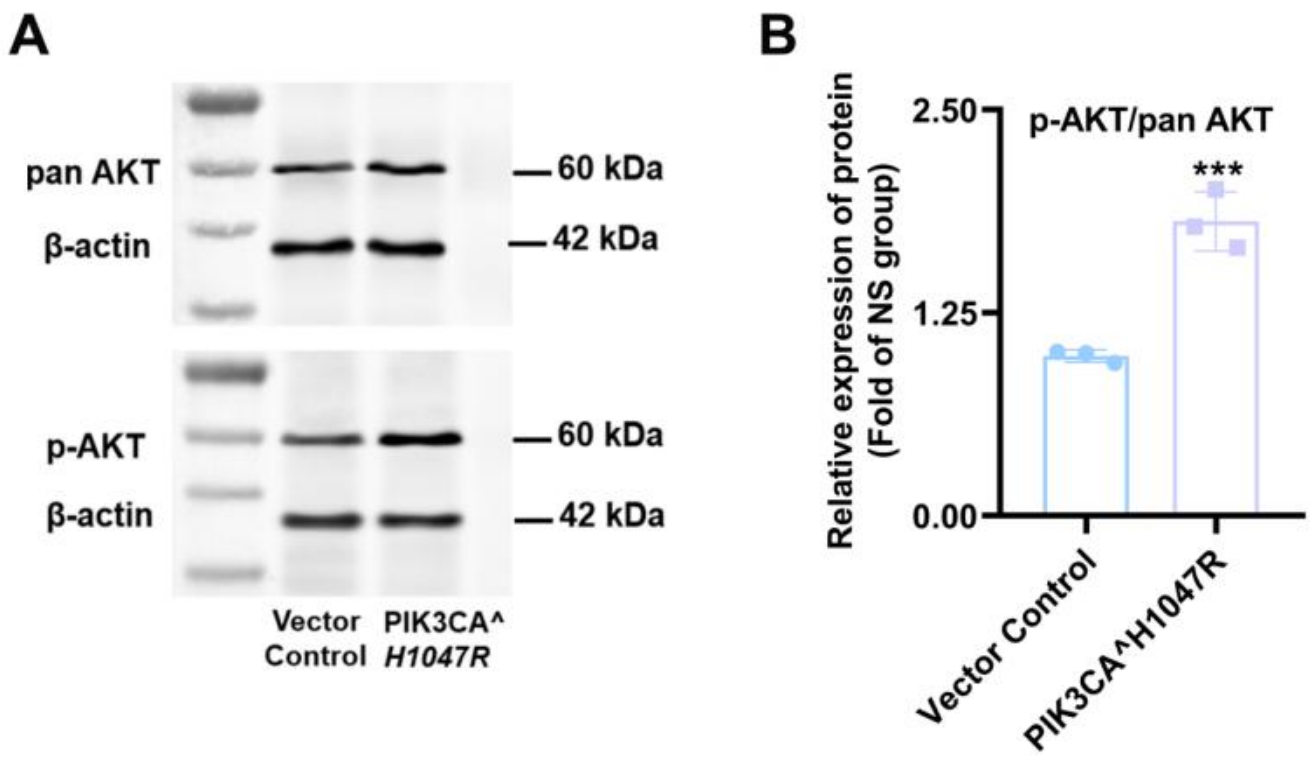

Fig. S2. Representative flow cytometry results of cell cycle detection.

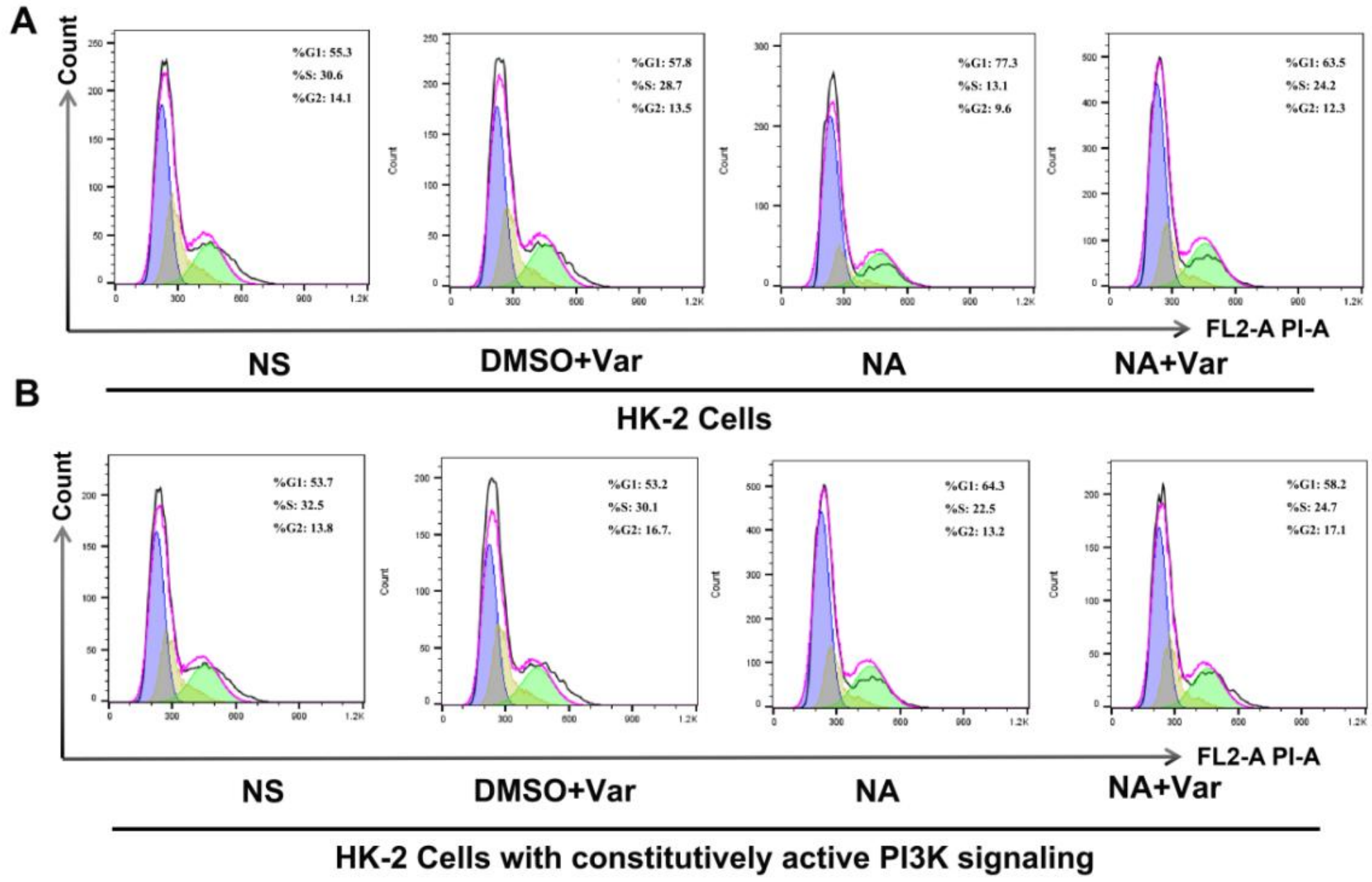

Fig. S3. *N. atra* SVPLA$_2$ impairs macrophage efferocytosis.

(A-D) Flow cytometric efferocytosis assays in BMDMs and RAW 264.7 cells, showing reduced uptake of apoptotic cells following venom exposure and rescue by varespladib.

All data are shown as mean ± SD. Significance levels: * $p < 0.05$, ** $p < 0.01$, and *** $p < 0.001$. ns denotes not significant versus the specified group indicated group. n = 3 biological replicates (in vitro).

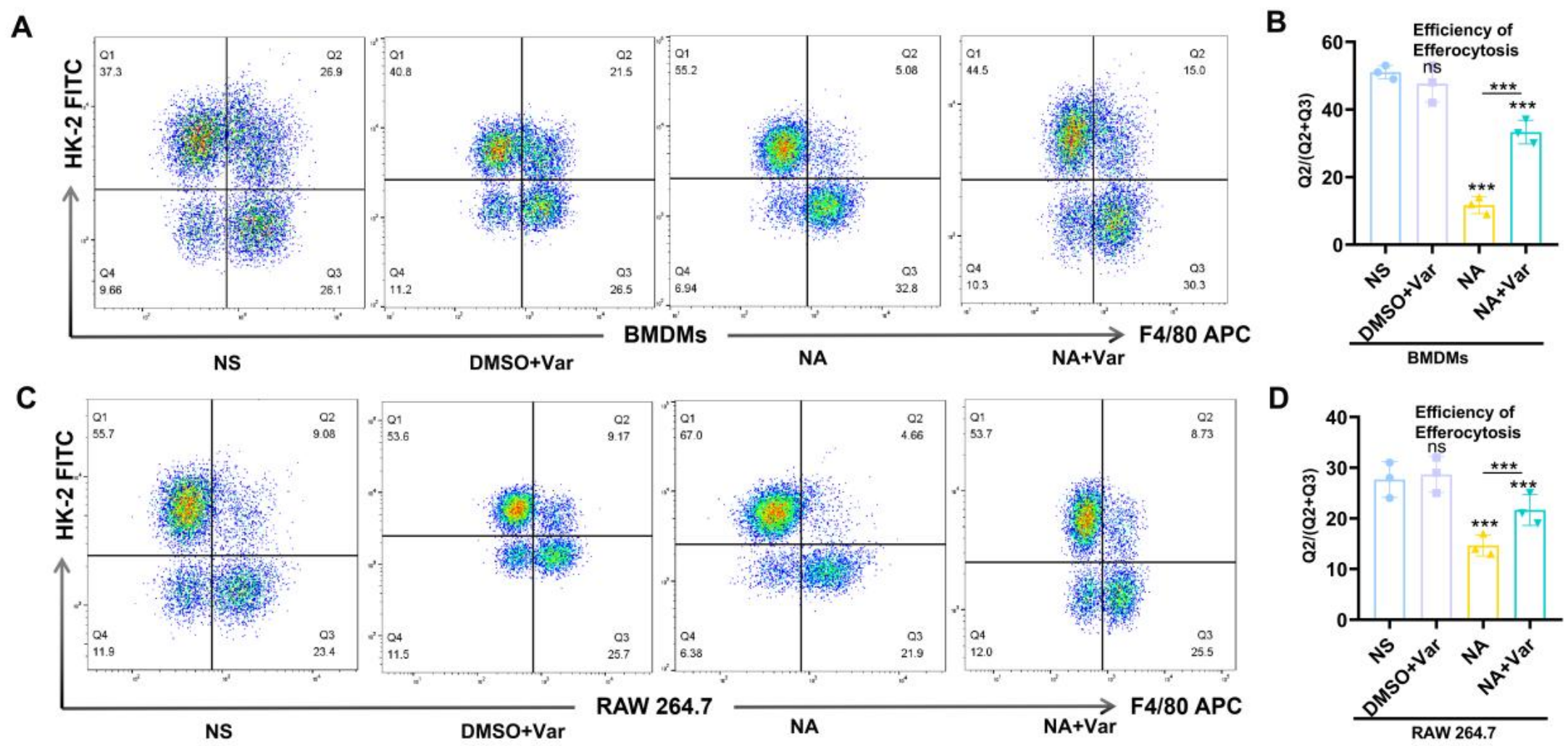

Fig. S4. Principal component analysis (PCA) plot of non-targeted metabolomics analysis.

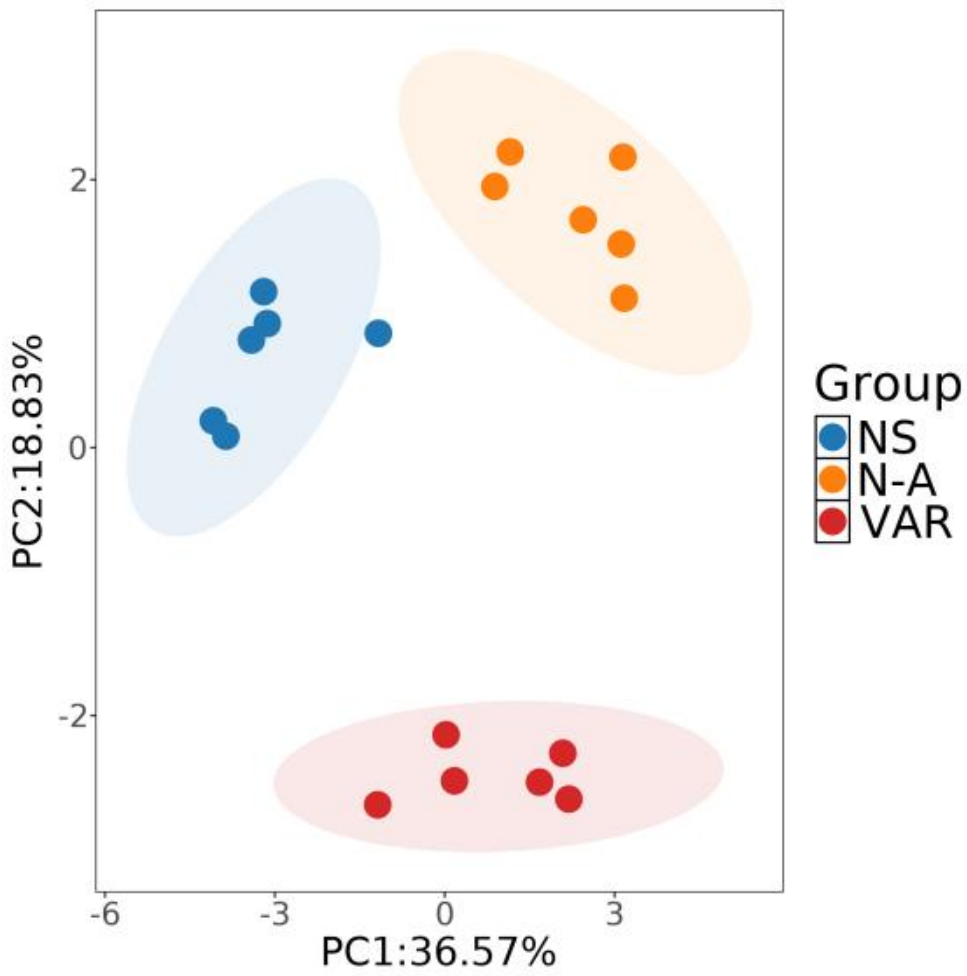

Fig. S5. Validation of HK2 knockdown efficiency in BMDMs.

(A) RT–qPCR analysis of HK2 mRNA expression in BMDMs transfected with control siRNA or HK2-specific siRNAs.

(B) Representative immunoblot analysis of HK2 protein expression following HK2 knockdown. β-actin was used as a loading control.

All data are shown as mean ± SD. Significance levels: * $p < 0.05$, ** $p < 0.01$, and *** $p < 0.001$. ns denotes not significant versus the specified group indicated group. n = 3 biological replicates (in vitro).

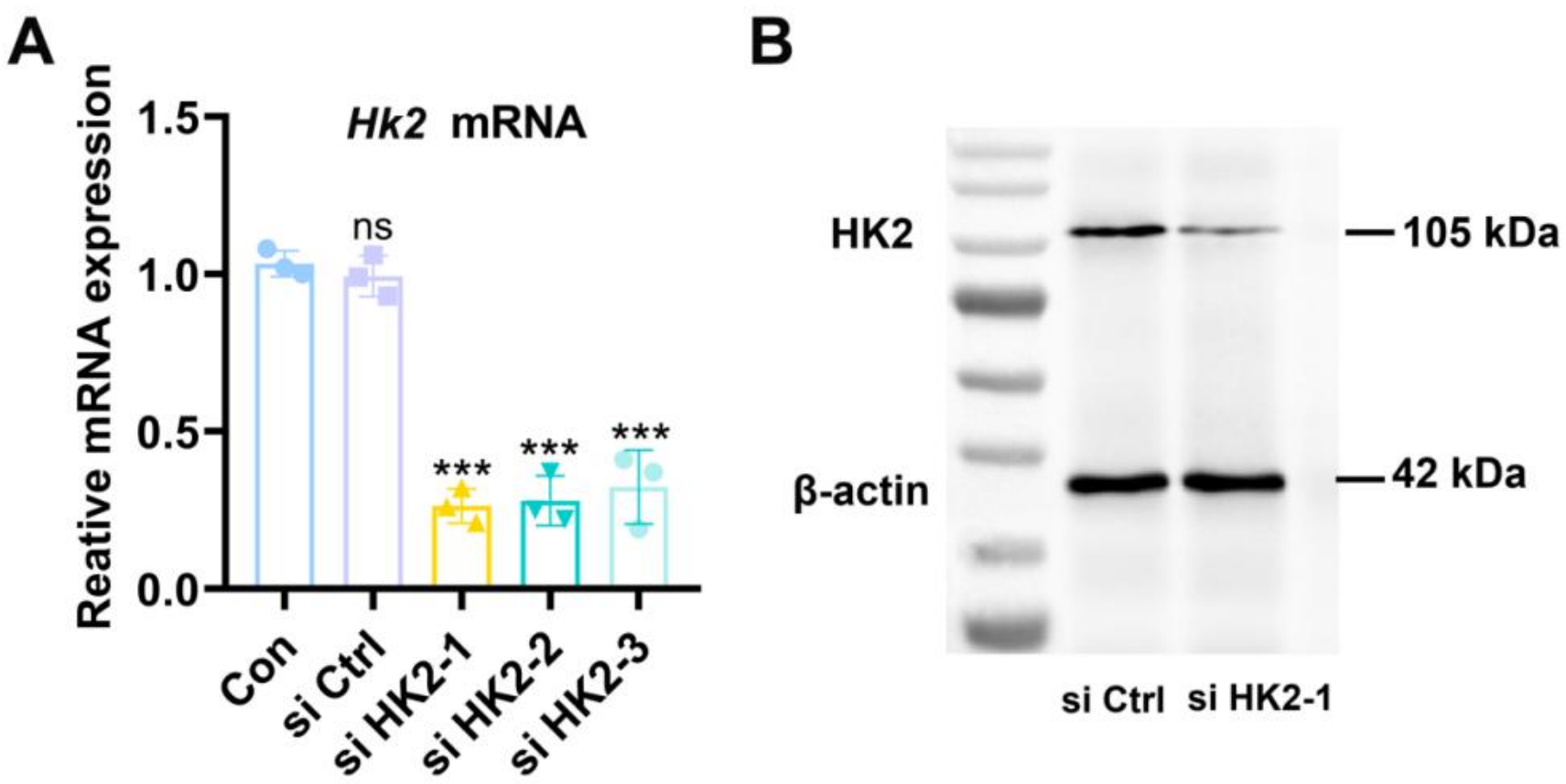

Supplement: Supplementary file 1 [file mmc1.pdf]
