# Supplementary material for: Naja atra SVPLA2 contributes to venom-induced AKI via HRAS palmitoylation-dependent PI3K-AKT inactivation
Source: J Lipid Res. 2026 Aug 14;67(9):101123. doi: 10.1016/j.jlr.2026.101123 (PMC13579411; doi:10.1016/j.jlr.2026.101123)
Supplement: Supplementary file 2 [file mmc2.pdf]

- **The representative western blot membranes in this study.**

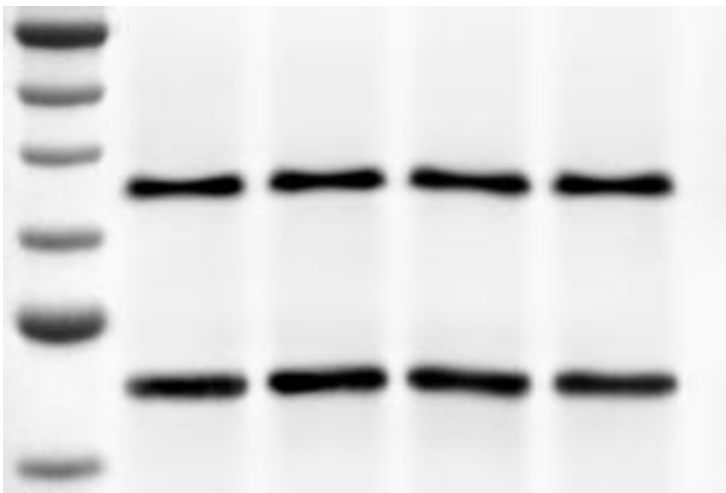

Full and uncropped western blots for Figure 3B

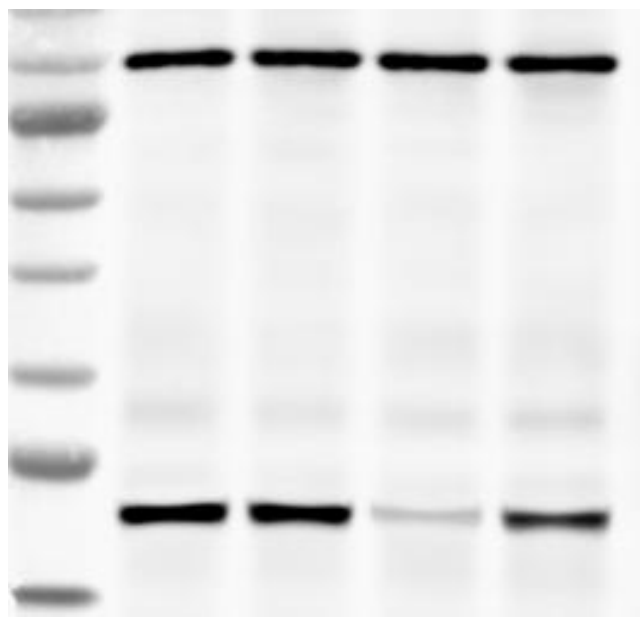

Full and uncropped western blots for Figure 3D

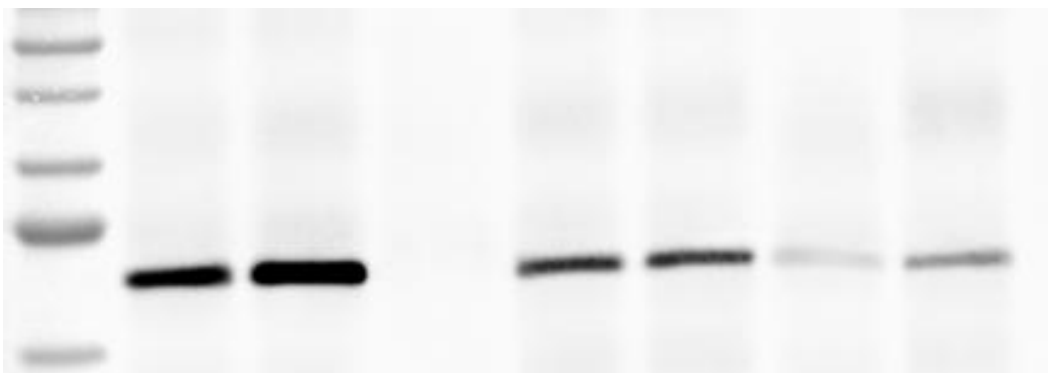

Full and uncropped western blots for Figure 3F

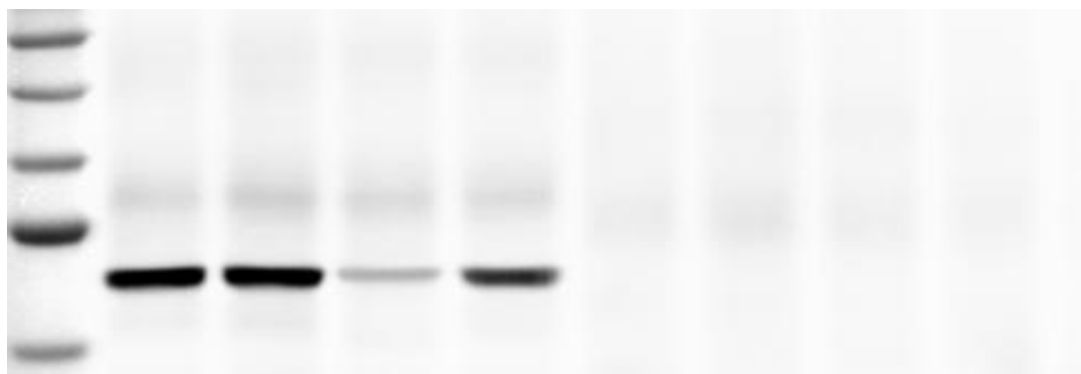

Full and uncropped western blots for Figure 3H

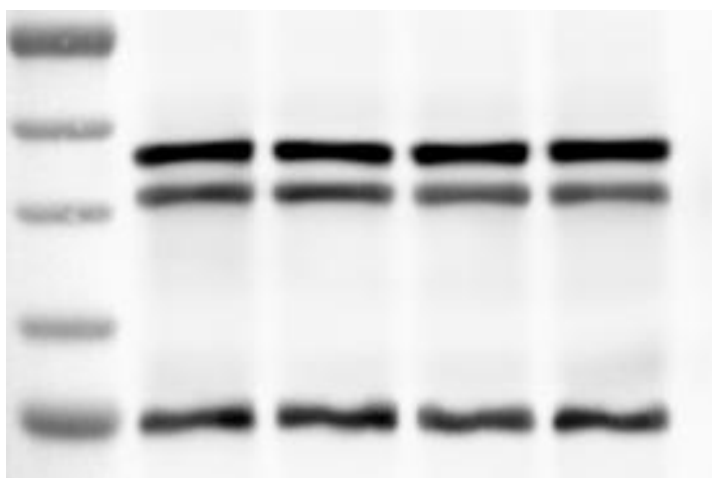

Full and uncropped western blots for Figure 3M

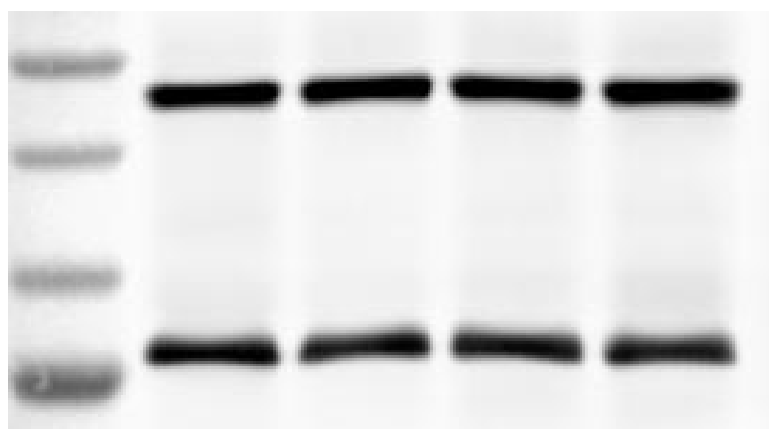

Full and uncropped western blots for Figure 3N

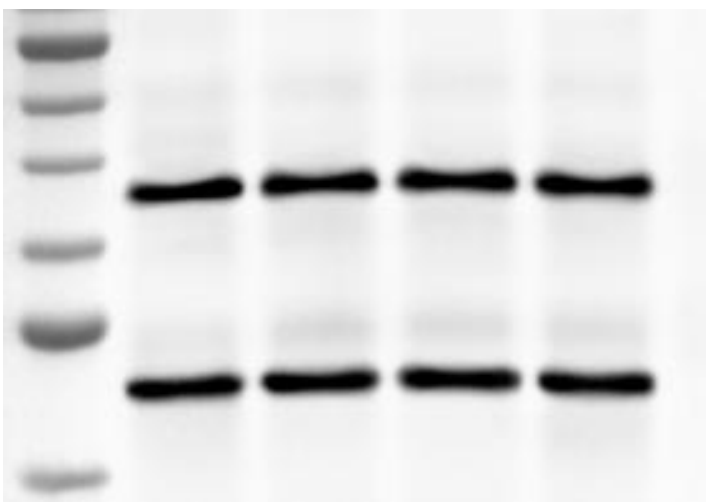

Full and uncropped western blots for Figure 3R

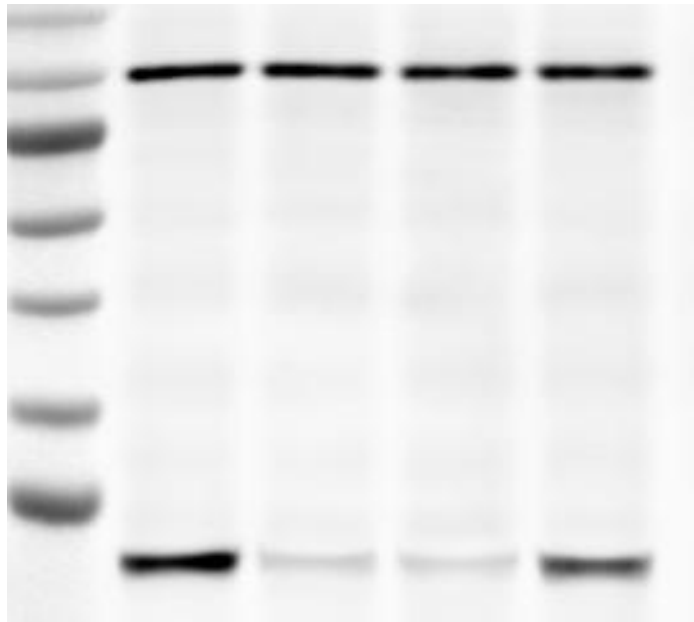

Full and uncropped western blots for Figure 3T

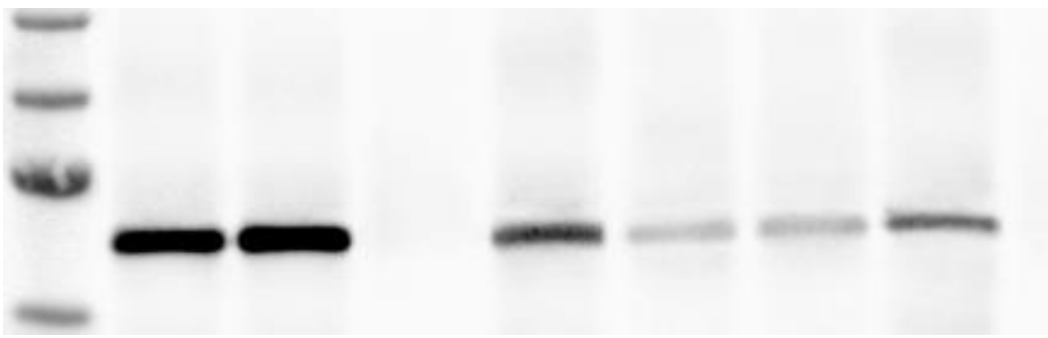

Full and uncropped western blots for Figure 3V

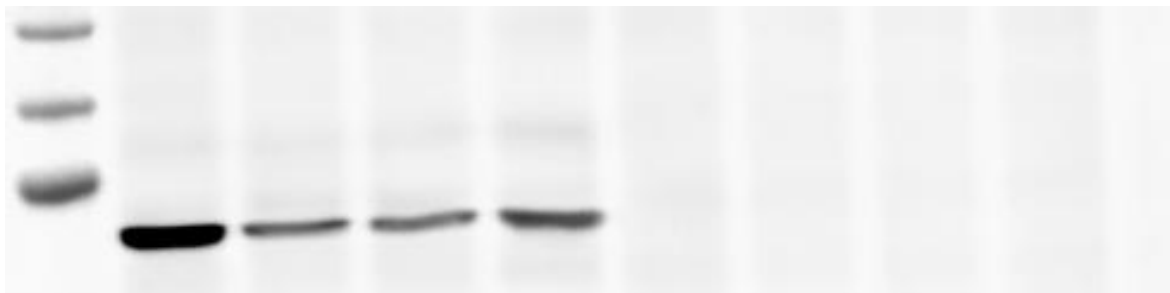

Full and uncropped western blots for Figure 3X

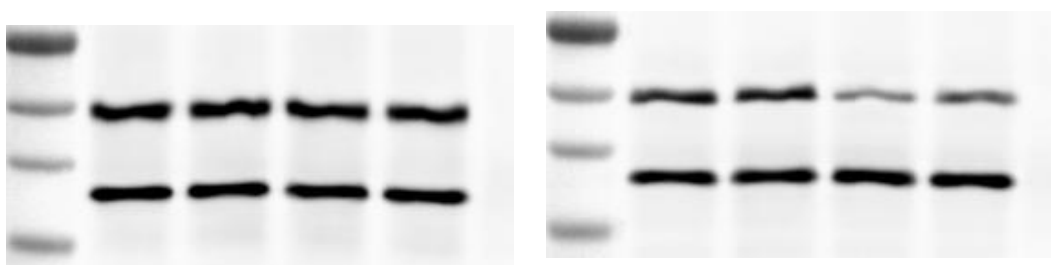

Full and uncropped western blots for Figure 4A

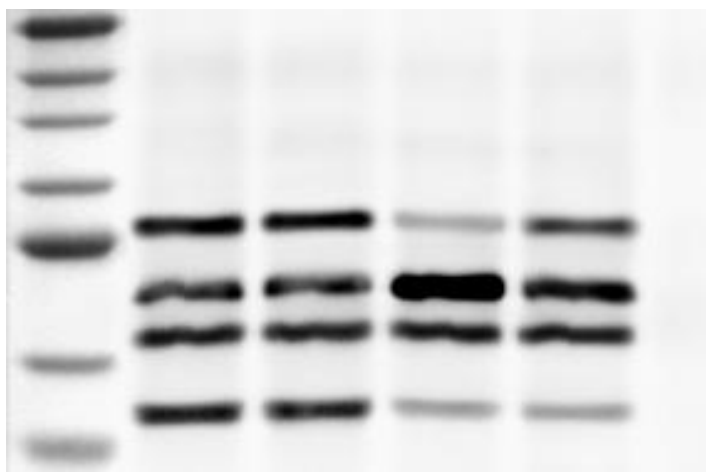

Full and uncropped western blots for Figure 4C

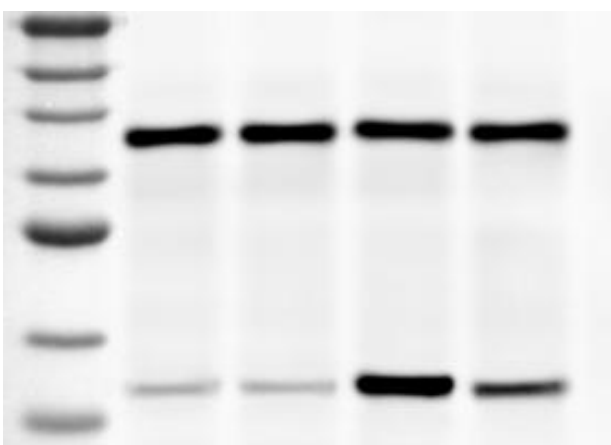

Full and uncropped western blots for Figure 4E

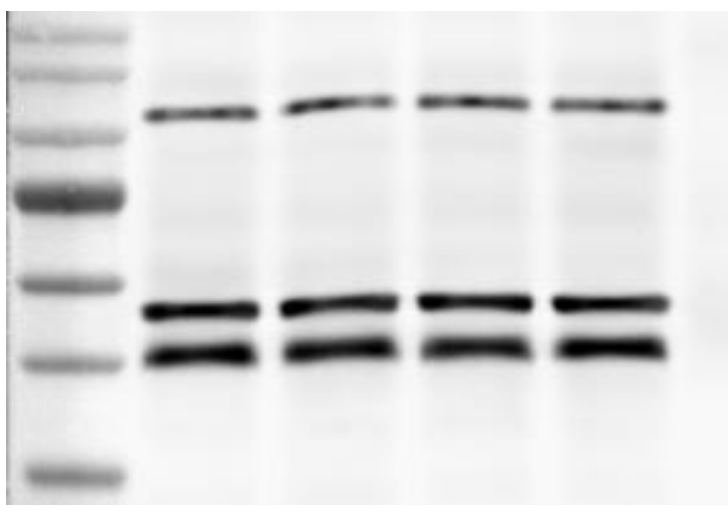

Full and uncropped western blots for Figure 4K

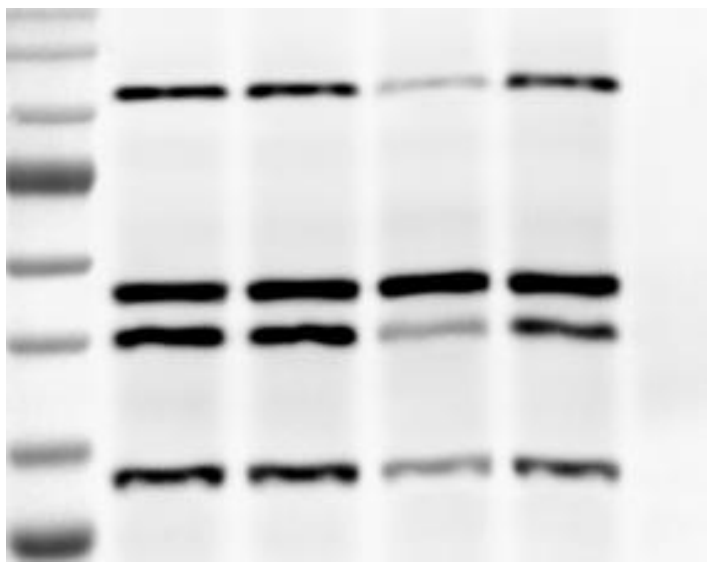

Full and uncropped western blots for Figure 4L

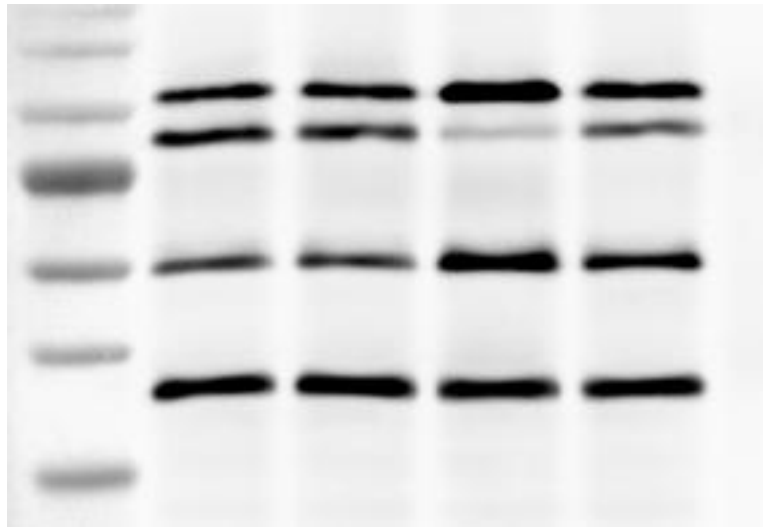

Full and uncropped western blots for Figure 7I

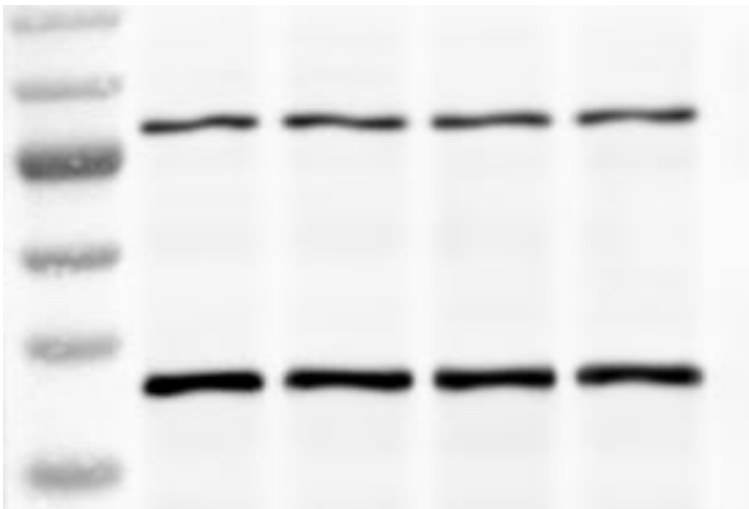

Full and uncropped western blots for Figure 7J

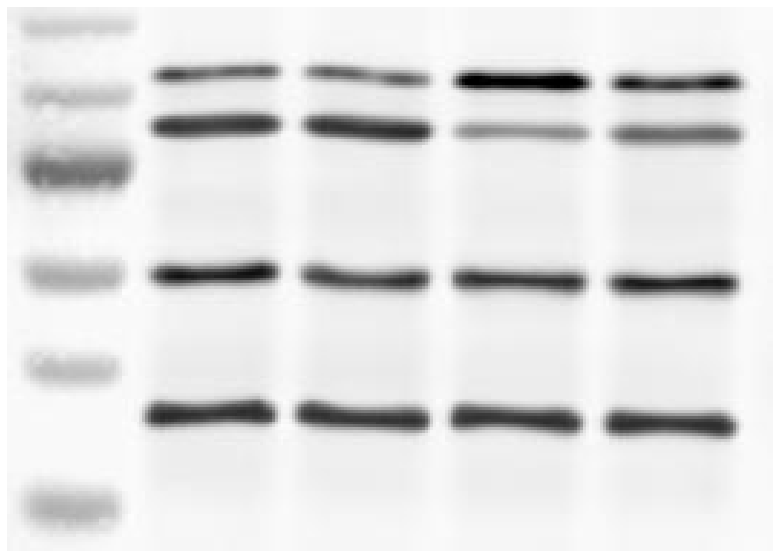

Full and uncropped western blots for Figure 7O

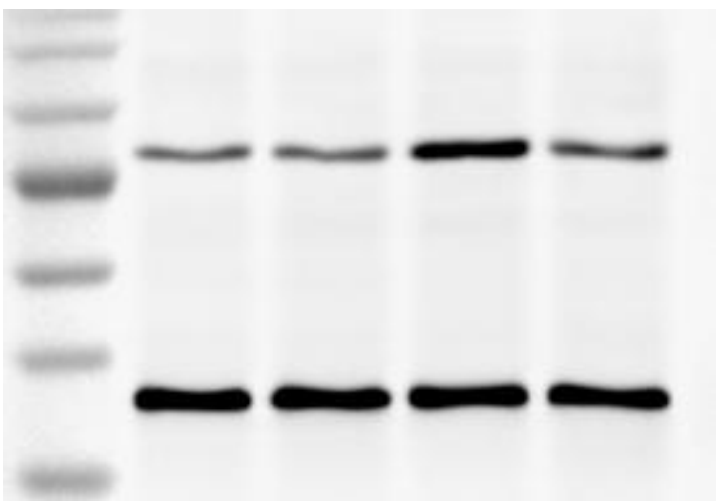

Full and uncropped western blots for Figure 7P

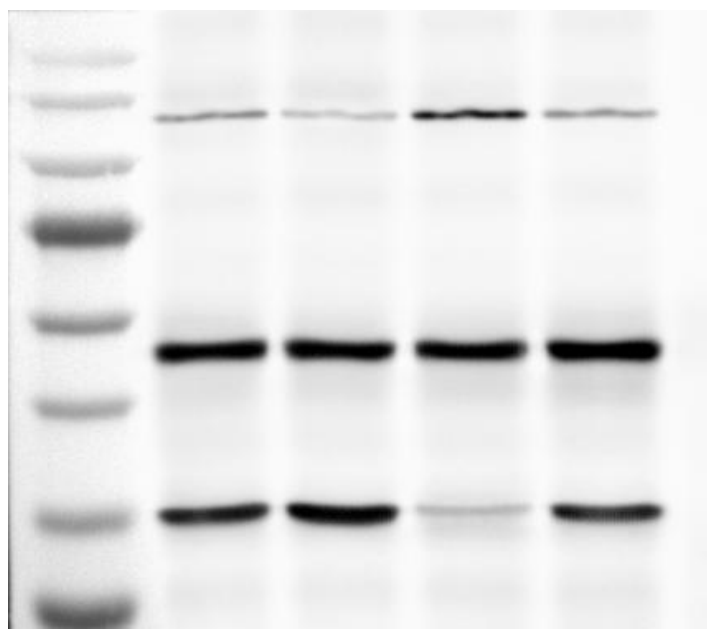

Full and uncropped western blots for Figure 8E
